# Human mesenchymal stromal cells transplanted into mice stimulate renal tubular cells and enhance mitochondrial function

**DOI:** 10.1038/s41467-017-00937-2

**Published:** 2017-10-17

**Authors:** Luca Perico, Marina Morigi, Cinzia Rota, Matteo Breno, Caterina Mele, Marina Noris, Martino Introna, Chiara Capelli, Lorena Longaretti, Daniela Rottoli, Sara Conti, Daniela Corna, Giuseppe Remuzzi, Ariela Benigni

**Affiliations:** 1 IRCCS—Istituto di Ricerche Farmacologiche Mario Negri, 24126 Bergamo, Italy; 2 Laboratory of Cell Therapy “G. Lanzani”, Azienda Socio Sanitaria Territoriale (ASST) Papa Giovanni XXIII, 24127 Bergamo, Italy; 3 Unit of Nephrology and Dialysis, Azienda Socio Sanitaria Territoriale (ASST) Papa Giovanni XXIII, 24127 Bergamo, Italy; 4 University of Milan, 20122 Milan, Italy

## Abstract

Mesenchymal stromal cells (MSCs) are renoprotective and drive regeneration following injury, although cellular targets of such an effect are still ill-defined. Here, we show that human umbilical cord (UC)-MSCs transplanted into mice stimulate tubular cells to regain mitochondrial mass and function, associated with enhanced microtubule-rich projections that appear to mediate mitochondrial trafficking to create a reparative dialogue among adjacent tubular cells. Treatment with UC-MSCs in mice with cisplatin-induced acute kidney injury (AKI) regulates mitochondrial biogenesis in proximal tubuli by enhancing PGC1α expression, NAD^+^ biosynthesis and Sirtuin 3 (SIRT3) activity, thus fostering antioxidant defenses and ATP production. The functional role of SIRT3 in tubular recovery is highlighted by data that in SIRT3-deficient mice with AKI, UC-MSC treatment fails to induce renoprotection. These data document a previously unrecognized mechanism through which UC-MSCs facilitate renal repair, so as to induce global metabolic reprogramming of damaged tubular cells to sustain energy supply.

## Abstract (web summary)

Mesenchymal stromal cells drive renal regeneration following injury. Here, the authors show that human mesenchymal stromal cells, when transplanted into mice with acute kidney injury, stimulate renal tubular cell growth and enhance mitochondrial function via SIRT3.

## Introduction

Mammalian kidneys, unlike those of fish and amphibians, have a limited capacity to repair, which becomes apparent when the damage is structurally and functionally confined to a small portion of the nephron^1, 2^. A meaningful example of the capacity of the mammalian kidney to regenerate is offered by the exuberant tubular cell proliferation that occurs during recovery from acute kidney injury (AKI)^1^. Advances in regenerative medicine have supported this paradigm, documenting in a convincing way that therapy with mesenchymal stromal cells (MSCs) can accelerate the kidney repair program after acute injury. This phenomenon is independent of MSC differentiation in the kidney but likely linked to paracrine effects of infused stromal cells on renal resident cells^3, 4^. Thus, results from studies in several experimental models of AKI have shown that treatments with rodent and human MSCs of different origins have an amazing protective effect on renal function impairment and structural damage, by reducing apoptosis and activating tubular cell turnover^5–9^. These renoprotective effects are linked to the MSC capacity to migrate to the site of renal damage and to release extracellular vesicles and pro-survival, anti-inflammatory, and immunomodulatory factors locally^5–9^. However, the precise intracellular renal targets responsible for the observed regenerative effects of MSC therapy have not been fully identified and conclusive mechanistic studies are still lacking. This is a critical issue, given that, sooner or later, clinical studies will be designed to give MSCs to patients with acute and even chronic renal dysfunction, with the aim of enhancing the regenerative capacity of the kidney. This has already been done to some extent, and the results are not always easy to interpret^10^. Hence, further investigations are needed to fully uncover the therapeutic potential of MSCs and to promote their safe use in humans.

The starting point for our present study is the observation that mitochondria dysregulation is a common early event preceding cell functional loss and death. Of all the nephron segments, the proximal tubular epithelium is endowed with the highest mitochondrial density due to its high-energy functions in active transport^11–13^. Tubular cells are the major targets of AKI, in which mitochondrial fission is coupled to membrane depolarization and permeabilization, with the release of apoptogenic factors associated with radical oxygen species (ROS) generation^11, 14^. The impairment of mitochondrial structural integrity ultimately results in ATP depletion and cytoskeletal changes, leading to the breakdown of the brush border, loss of cell–cell contact, and tubular epithelial cell detachment^11–16^. Microtubules, one of the primary components of the cytoskeleton, have been described to regulate intracellular mitochondrial distribution^17–19^. Together, the dysregulation of both functional and structural integrity of mitochondria is the critical early event responsible for tissue injury occurring during AKI and the progression of the disease^11, 14, 20^.

Several studies have discovered that different mitochondrial processes such as energy production^21, 22^ and antioxidant defences^23^ are critically dependent on Sirtuin 3 (SIRT3) due to its deacetylase activity^24^. We have previously documented that extended lifespan in mice is associated with reduced oxidative damage, increased mitochondrial number, and the upregulation of SIRT3 in the kidney^25^. In line with this evidence, SIRT3 downregulation was associated with the development of age-associated disorders such as metabolic syndrome^26^. More recently we also uncovered the role of SIRT3 as a master regulator of injury and repair through the preservation of mitochondrial dynamics in AKI^20, 27^. Pharmacological manipulations with agents able to restore renal SIRT3 levels and impaired mitochondrial dynamics ultimately resulted in kidney repair in the AKI animals^20^.

With this background, the aim of this study was to investigate whether the effects of human umbilical cord-derived (UC) MSCs to accelerate renal repair in rodents were dependent on the restoration of SIRT3 expression and activity, thus regulating mitochondrial function and distribution in the proximal tubular cell compartment. Here, we report a mechanism through which cell-based therapy induced kidney repair by enhancing SIRT3, thus preserving mitochondria functional integrity, a condition that is indispensable for the microtubule-dependent organelle trafficking among injured tubular epithelial cells to restore their bioenergetic profile during AKI.

## Results

### UC-MSCs foster repair and replenish mitochondria in AKI

**Fig. 1:**
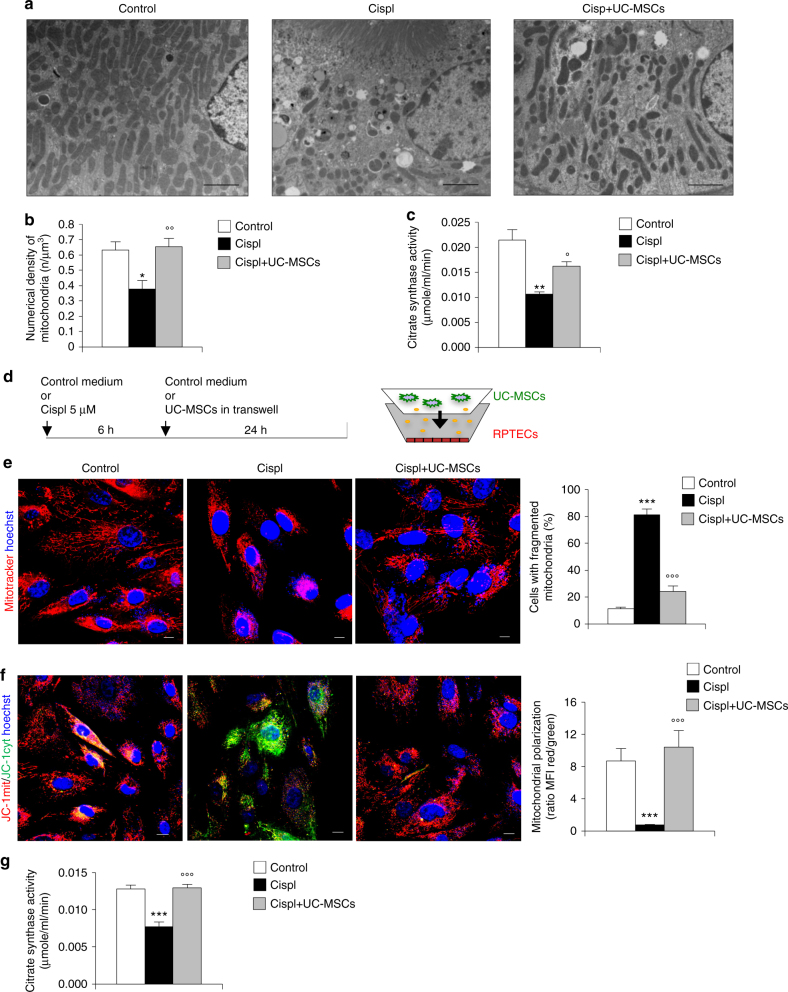
Human UC-MSCs preserve mitochondria integrity both in mouse renal tissue and in cultured tubular cells after cisplatin injury. **a** Representative transmission electron micrographs showing fragmentation of mitochondria into short rods in proximal tubular cells of a cisplatin mouse given saline (Cispl), in respect to a control mouse at 4 days. These ultrastructural alterations were normalized by UC-MSC treatment. Scale bar 2000 nm. **b** Morphometric analysis of the number of mitochondria per volume (n/µm^3^) in proximal tubular cells of control (*n* = 4 mice) and cisplatin mice given saline (*n* = 5 mice) or UC-MSCs (*n* = 6 mice) at 4 days. *P < 0.05 vs Control and °°*P* < 0.01 vs Cispl using ANOVA corrected with Bonferroni coefficient. **c** Citrate synthase activity, marker of mitochondrial mass, assessed in kidneys of control and cisplatin mice given saline or UC-MSCs at 4 days (*n* = 4 mice per group). **P < 0.01 vs Control and °*P* < 0.05 vs Cispl using ANOVA corrected with Bonferroni coefficient. **d** Schematic representation of the experimental design and the transwell system used for in vitro experiments. **e** Representative images and quantification of mitochondrial fragmentation visualized by MitoTracker staining (red) in control RPTECs, cisplatin-treated RPTECs alone or co-cultured with UC-MSCs (*n* = 3 independent experiments). Nuclei are counterstained with hoechst (blue). Scale bar 10 μm. ***P < 0.001 vs Control and °°°*P* < 0.001 vs Cispl using ANOVA corrected with Bonferroni coefficient. **f** Representative images and quantification of mitochondrial membrane potential (red/green florescent area) visualized by JC-1, a dye sensitive to mitochondrial membrane potential (ΔΨm) changes that shifts the emission spectrum from red (mitochondrial distribution, JC-1mit) to green (cytoplasmic distribution, JC-1cyt) in control RPTECs, cisplatin-treated RPTECs alone or co-cultured with UC-MSCs (*n* = 3 independent experiments). Nuclei are counterstained with hoechst (blue). Scale bar 10 μm. ***P < 0.001 vs Control and °°°*P* < 0.001 vs Cispl using ANOVA corrected with Bonferroni coefficient. **g** Citrate synthase activity, reflecting mitochondrial mass, assessed in control RPTECs, cisplatin-treated RPTECs alone or co-cultured with UC-MSCs (*n* = 4 independent experiments). ***P < 0.001 vs Control and °°°*P* < 0.001 vs Cispl using ANOVA corrected with Bonferroni coefficient. Data in **b**, **c** and **e**–**g** are expressed as mean ± SEM

Immunodeficient mice injected with cisplatin developed severe renal function impairment (Supplementary Fig. 1A) and proximal tubular injury consisting of hyaline casts, tubular cell degeneration, and necrosis at 4 days (Supplementary Fig. 1B, C). The intravenous administration of human UC-MSCs 1 day after cisplatin treatment exerted a renoprotective effect to the extent that renal function, evaluated as blood urea nitrogen (BUN), improved and signs of tubular damage were reduced (Supplementary Fig. 1A–C). Transplanted human UC-MSCs engrafted injured mouse renal tissues with a frequency averaging 1.6 ± 0.26 human nuclear antigen (HNA)-positive MSCs/10^5^ renal cells and distributed predominantly in the peritubular areas (Supplementary Fig. 1D, E). Electron microscopy micrographs of proximal tubuli in cisplatin mice revealed that mitochondria fragmented into short rods or spheres, unlike the elongated organelles observed in control tissues (Fig. 1a). Morphometric analysis also indicated that mitochondria rarefied in cisplatin mice compared to controls (Fig. 1b). Treatment with UC-MSCs normalized mitochondrial shape (Fig. 1a) and density (Fig. 1b). There was a consistent and important decrease in the mitochondrial mass—assessed as citrate synthase activity—in tubular epithelial cells of AKI mice, which was restored by UC-MSC infusion (Fig. 1c). All the above beneficial effects were specific for UC-MSCs, since human fibroblast infusion did not protect mice with cisplatin-induced AKI from renal functional and structural impairment, and mitochondrial fragmentation (Supplementary Fig. 2A–C)

### UC-MSCs preserve mitochondria in tubular cells

We set up an in vitro model of tubular cell injury by using human renal proximal tubular epithelial cells (RPTECs) exposed to cisplatin and then co-cultured with or without UC-MSCs in a transwell system (see scheme in Fig. 1d). As it occurs in vivo^6, 8, 28^, cisplatin exerted its cytotoxic effect by reducing the number of living RPTECs (Supplementary Fig. 3A) and by inhibiting their proliferation at 24 h (Supplementary Fig. 3B). The expression of the apoptotic marker cleaved caspase-3 was increased 24 h after cisplatin exposure compared to resting cells (Supplementary Fig. 3C). Co-culture with UC-MSCs increased tubular cell number and proliferation, and reduced the apoptotic cells (Supplementary Fig. 3A–C).

Since tubular injury is closely associated with mitochondrial dysfunction^11–14^, we examined the functional and structural integrity of mitochondria. Cisplatin-damaged RPTECs showed massive mitochondrial fragmentation as early as at 6 h (Supplementary Fig. 3D), which was still detected at 24 h (Fig. 1e) when 81% of cisplatin-exposed RPTECs exhibited round-shaped fragmented mitochondria compared with the filamentous network of elongated mitochondria of control cells. Then, we assessed mitochondrial membrane potential (Δψ_m_) using the potential-sensor JC-1, which shifts from green to red in the emission spectrum, reflecting cytoplasmic vs mitochondrial distribution. Cisplatin induced massive tubular mitochondrial depolarization, as evidenced by diffuse cytoplasmic JC-1 at 6 h (Supplementary Fig. 3E), which was also stably recorded after 24 h (Fig. 1f). The co-culture of cisplatin-treated RPTECs with UC-MSCs restored tubular mitochondrial dynamics markedly by reducing fragmentation (Fig. 1e) and normalizing RPTEC mitochondrial membrane potential (Fig. 1f) at 24 h. Consistently, a reduction in mitochondrial mass was observed in cisplatin-treated RPTECs at 24 h, which was reversed by the UC-MSCs (Fig. 1g), suggesting that mitochondria are critical targets of MSC regenerative activity. At variance, co-culture of injured RPTECs with human fibroblasts, here used as control cells, did not result in amelioration of mitochondrial structural and functional integrity (Supplementary Fig. 2D–G).

### UC-MSCs induce tubular cell transcriptional reprogramming

**Fig. 2:**
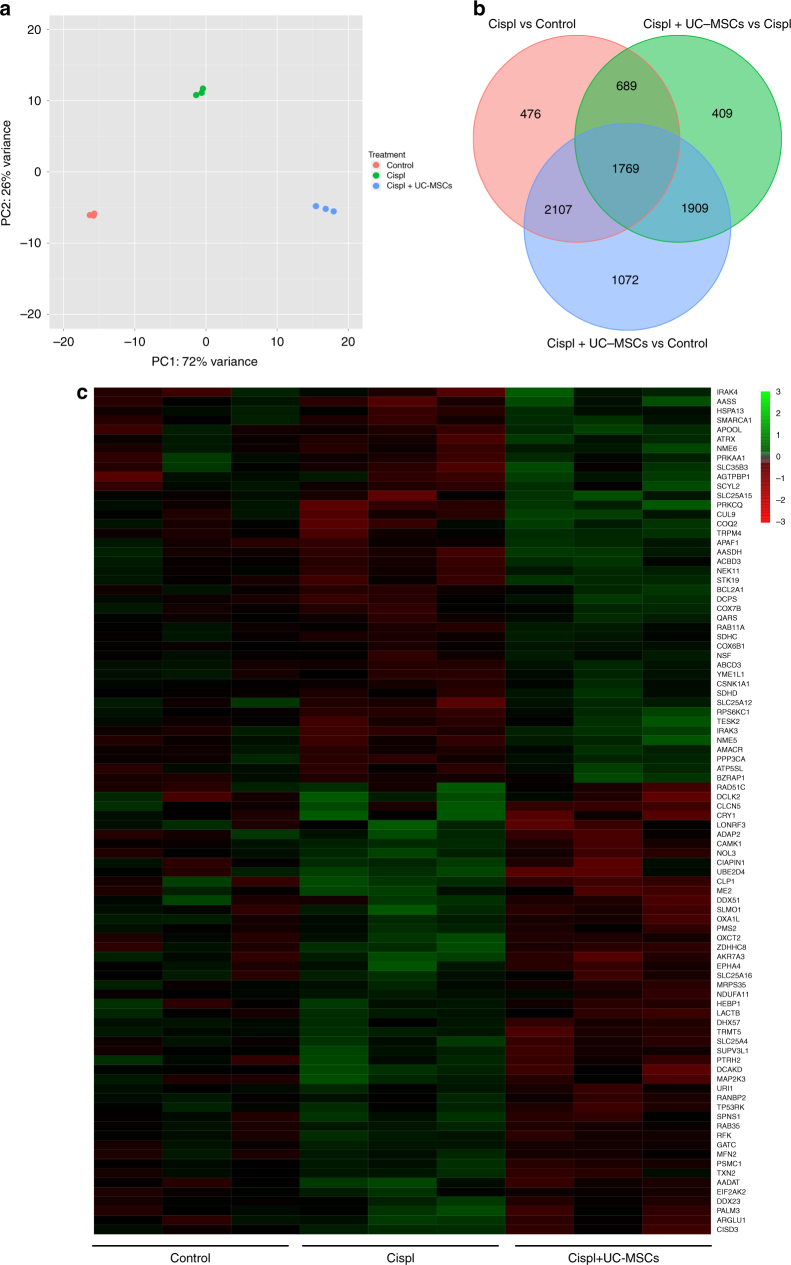
Human UC-MSCs induce a global transcriptional reprogramming in cisplatin-damaged RPTECs. **a** Principal component analysis (PCA) on rlog transformed read counts, in which the three RPTEC experimental groups (Control, Cispl, and Cispl + UC-MSCs) were separated along the axis of major variation (PC1, 72% of variance). **b** Overlapping of the differentially expressed genes (DEGs) in the three comparisons: Cispl + UC-MSCs vs Cispl, Cispl vs Control, and Cispl + UC-MSCs vs Control. **c** Clustering of genes related to mitochondrial energy homeostasis among the 409 uniquely DEGs in Cispl + UC-MSCs vs Cispl

To identify the genes involved in the protective effect of UC-MSCs on cisplatin-injured RPTECs in vitro, a genome-wide differential gene expression analysis was performed. Consistency among replicates was inspected visually, as shown in Supplementary Fig. 4A. There was high homogeneity for each experimental condition between the three replicates, with correlation coefficients above 0.99 for all pairwise comparisons. The homogeneity was confirmed by principal component analysis (PCA) on transformed read counts, where the three replicates clustered together for each RPTEC experimental group (Control, Cispl, and Cispl + UC-MSCs) (Fig. 2a). In addition, PCA showed that the three groups were separated along the axis of major variation (PC1, 72% of variance) indicating that the expression profiles were different in the three groups. Indeed, a striking difference in the gene expression profiles was found between the Control vs Cispl and Cispl vs Cispl + UC-MSC groups (Fig. 2a). Notably, the highest differential gene expression was observed in the Control vs Cispl + UC-MSCs group.

Differentially expressed gene (DEG) analysis showed that 4776, 5041, and 6857 genes were differentially expressed (at adjusted *P* < 0.05) in Cispl + UC-MSCs vs Cispl, Cispl vs Control, and Cispl + UC-MSCs vs Control, respectively (Fig. 2b). Top DEGs (|log_2_FC| > 2) for the Cispl + UC-MSCs vs Cispl comparison are listed in Supplementary Data 1 and 2. Of the enriched categories, chemotaxis together with proliferation, regulation of cell morphogenesis/differentiation, and response to hypoxia were the most strongly represented (Supplementary Fig. 4B).

We focussed on the 409 genes that were exclusively modulated by UC-MSC treatment in respect to cells treated with cisplatin alone and were not shared among the three comparisons (Fig. 2b; Supplementary Data 3), possibly representing a de novo regenerative response induced by UC-MSCs. Notably, in the enrichment analysis of these genes, the pathways related to mitochondria and energy homeostasis were strongly represented (Fig. 2c; Supplementary Fig. 5), including: lysine degradation and NADP(H) generation (*AAS*, aminoadipate-semialdehyde synthase), fatty acid metabolism (*AASDH*, aminoadipate-semialdehyde dehydrogenase), mitochondrial cholesterol transport (*ACBD3*, TSPO-associated acyl-coenzyme A binding domain containing), and urea cycle (*SLC25A* 12/15, solute carrier family 25—Aspartate/Glutamate—Member 12 and 15). Additionally, in cisplatin-injured RPTECs, UC-MSC treatment enhanced the gene expression of electron transport chain components (*COQ2*, coenzyme Q2 4-hydroxybenzoate polyprenyltransferase; *COX 6B1* and *7B*, cytochrome *c* oxidase 6B1 and 7B; *SDH C* and *D*, succinate dehydrogenase complex subunit C and D), and proteins involved in ATP production (*ATP5SL*, ATP synthase H+ transporting, mitochondrial Fo complex, subunit S like and *BZRAP1*, translocator protein formerly known as the peripheral benzodiazepine receptor). In addition, the energy sensor adenosine monophosphate-activated protein kinase (AMPK: *PRKAA1*) was also upregulated by UC-MSC treatment as indicated by the enrichment analysis (Fig. 2c; Supplementary Data 3). Moreover, the AMPK downstream target nicotinamide phosphoribosyltransferase (*NAMPT*)—the rate-limiting enzyme in the nicotinamide adenine dinucleotide (NAD^+^) salvage pathway^29^—was upregulated 5.8-fold (log_2_FC: 2.550) in the top DEGs between Cispl + UC-MSCs vs Cispl (Supplementary Data 1). Additionally, kynureninase (*KYNU*)—involved in the biosynthetic pathway of NAD^+^ from tryptophan catabolism^30^—was 15-fold upregulated (log_2_FC: 3.91) in Cispl + UC-MSCs vs Cispl (Supplementary Data 1).

### UC-MSCs normalize cell energy and mitochondrial acetylome

**Fig. 3:**
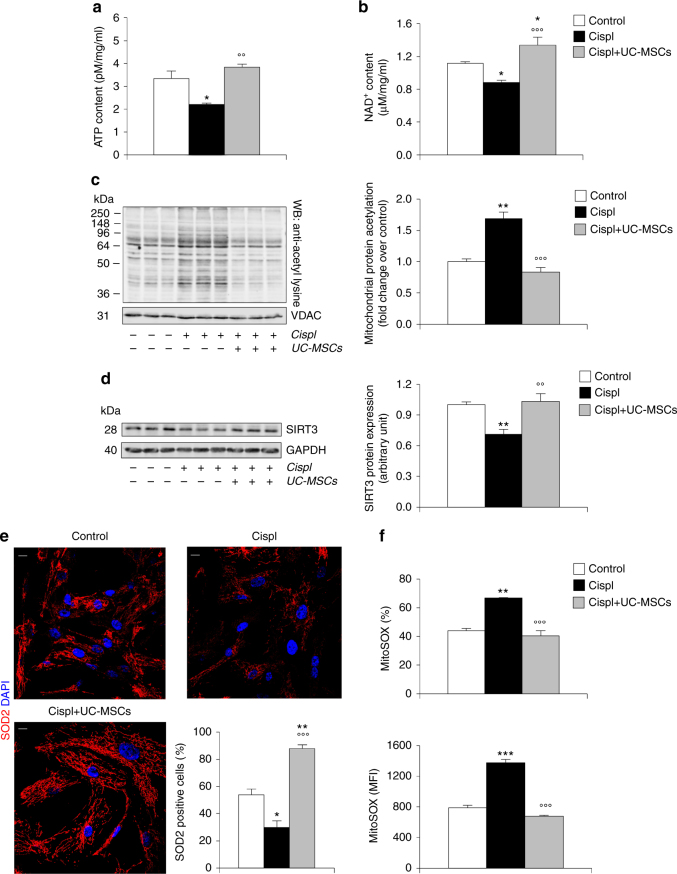
Human UC-MSCs regulate SIRT3 mitochondrial targets involved in the recovery of cisplatin-injured RPTECs. **a** Evaluation of ATP content in control RPTECs, cisplatin-treated RPTECs alone or co-cultured with UC-MSCs (*n* = 3 independent experiments). *P < 0.05 vs Control and °°*P* < 0.01 vs Cispl using ANOVA corrected with Bonferroni coefficient. **b** Evaluation of NAD^+^ content in control RPTECs, cisplatin-treated RPTECs alone or co-cultured with UC-MSCs (*n* = 6 independent experiments). *P < 0.05 vs Control and °°°*P* < 0.001 vs Cispl using ANOVA corrected with Bonferroni coefficient. **c** Representative western blots and densitometric analysis of protein acetylation in mitochondria isolated from control RPTECs, cisplatin-treated RPTECs alone or co-cultured with UC-MSCs (*n* = 3 independent experiments). Molecular weights (kDa) are shown on the left. **P < 0.01 vs Control and °°°*P* < 0.001 vs Cispl using ANOVA corrected with Bonferroni coefficient. **d** Representative western blots and densitometric analysis of SIRT3 protein expression in control RPTECs, cisplatin-treated RPTECs alone, or co-cultured with UC-MSCs (*n* = 6 independent experiments). **P < 0.01 vs Control and °°*P* < 0.01 vs Cispl using ANOVA corrected with Bonferroni coefficient. **e** Representative images and quantification of SOD2 (red) protein expression by immunofluorescence analysis in control RPTECs, cisplatin-treated RPTECs alone or co-cultured with UC-MSCs (*n* = 3 independent experiments). Nuclei are counterstained with DAPI (blue). Scale bar 10 μm. *P < 0.05, **P < 0.01 vs Control, and °°°*P* < 0.001 vs Cispl using ANOVA corrected with Bonferroni coefficient. **f** Evaluation of mitochondrial superoxide generation by MitoSOX fluorescent probe and expressed as both the percentage of ROS producing cells (%, upper panel) and the mean fluorescence intensity (MFI, lower panel) in control RPTECs, cisplatin-treated RPTECs alone or co-cultured with UC-MSCs (*n* = 3 independent experiments). **P < 0.01, ***P < 0.001 vs Control and °°°*P* < 0.001 vs Cispl using ANOVA corrected with Bonferroni coefficient. Data in **a**–**f** are expressed as mean ± SEM

Given the high representation of DEGs involved in mitochondrial energy metabolism and NAD^+^ biosynthesis, we evaluated the effect in vitro of UC-MSCs on ATP and NAD^+^ content in injured RPTECs. A decrease in total ATP was observed in cisplatin-injured RPTECs, while ATP content was enhanced by UC-MSCs in injured tubular cells (Fig. 3a). We also found a significant decrease in total NAD^+^ cellular content in cisplatin-injured RPTECs compared to resting cells (Fig. 3b), which was replenished over control levels by UC-MSC treatment (Fig. 3b). Since NAD^+^ is a cofactor of the main mitochondrial deacetylase SIRT3^31, 32^, we evaluated the mitochondrial protein acetylation at the ɛ-amino group of lysine residues. Protein acetylation was increased greatly in mitochondria isolated from cisplatin-injured RPTECs, unlike in resting cells (Fig. 3c). Treatment with UC-MSCs decreased the protein-acetylated residues (Fig. 3c), indicating their ability to restore SIRT3 activity. SIRT3 messenger RNA (mRNA) and protein expression were consistently reduced (by 40% and 30%, respectively) in injured RPTECs compared to control cells and it recovered after treatment with UC-MSCs (Supplementary Fig. 6A; Fig. 3d).

Since SIRT3 regulates mitochondrial antioxidant activity^23^, we evaluated the expression of superoxide dismutase 2 (SOD2). As shown in Supplementary Data 1, a 6.6-fold increase (log_2_FC: 2.73) in SOD2 mRNA expression was found in the top DEGs by RPTECs exposed to Cispl + UC-MSCs compared to Cispl. The protein expression of SOD2 was reduced by cisplatin and was enhanced by UC-MSC treatment, even ﻿over﻿ control, as highlighted by a very high signal of SOD2-positive mitochondria in cell cytoplasm and projections (Fig. 3e). In parallel, cisplatin induced ROS increase in RPTECs, assessed by the O2^•−^ sensor MitoSOX, compared to untreated cells (Fig. 3f). Co-culture with UC-MSCs exerted a strong antioxidant effect (Fig. 3f).

### UC-MSCs promote mitochondrial exchange along microtubules

**Fig. 4:**
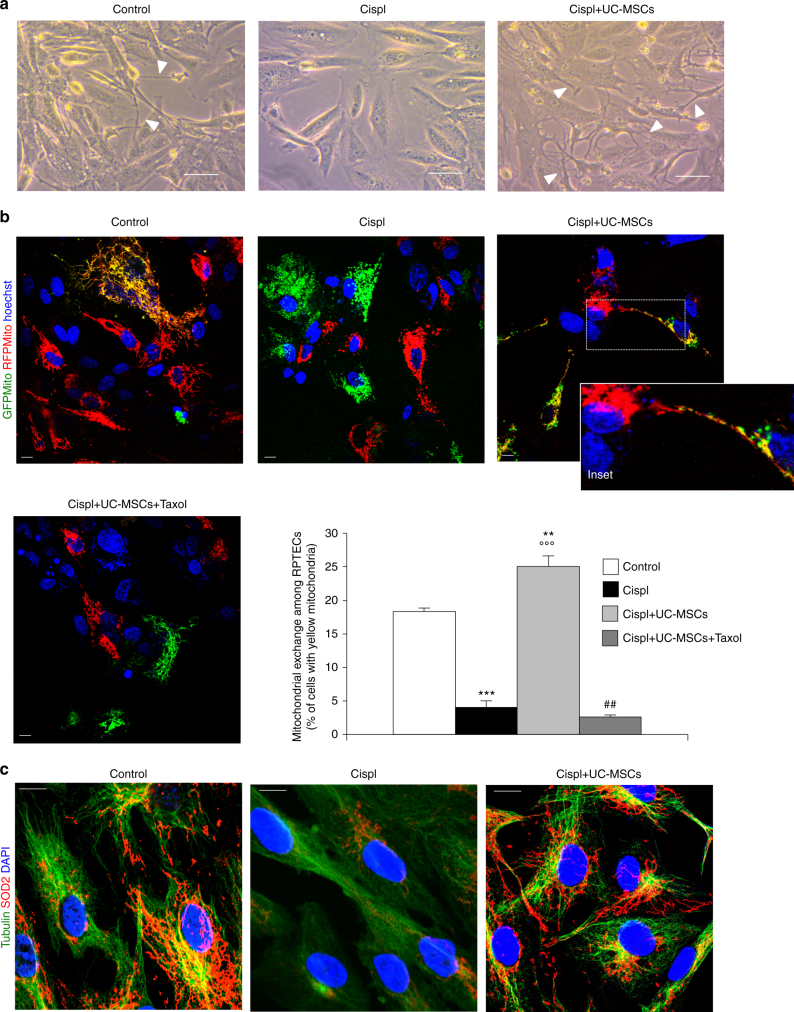
Human UC-MSCs favor mitochondrial exchange among injured RPTECs through microtubules. **a** Representative phase contrast images showing the morphology of control RPTECs, cisplatin-treated RPTECs alone, or co-cultured with UC-MSCs (*n* = 6 independent experiments). Cytoplasmic protrusions connecting adjacent tubular cells (indicated by arrowheads) markedly decreased in response to cisplatin and were normalized by UC-MSC exposure. Scale bar 10 μm. **b** Representative images and quantification of mitochondrial exchange in control RPTECs, cisplatin-treated RPTECs alone, or co-cultured with UC-MSCs (*n* = 4 independent experiments). Two different populations of cultured tubular cells were obtained by transfecting mitochondria with a fusion construct of E1 alpha pyruvate dehydrogenase leader sequence with GFP (green) or RFP (red) and the mitochondrial exchange was assessed as the co-localizing signals (yellow). Enlarged detail of the mitochondria trafficking among RPTECs through cytoplasmic protrusion is shown in inset. In additional samples, cisplatin-treated RPTECs were exposed to taxol in co-incubation with UC-MSCs (*n* = 4 independent experiments). Nuclei are counterstained with hoechst (blue). Scale bar 10 μm. **P < 0.01, ***P < 0.001 vs Control and °°°*P* < 0.001 vs Cispl and ##*P* < 0.01 vs Cispl + UC-MSCs using ANOVA corrected with Bonferroni coefficient. **c** Representative images of immunofluorescence analysis of tubulin network (green) and SOD2-labeled mitochondria (red) in control RPTECs, cisplatin-treated RPTECs alone or co-cultured with UC-MSCs (*n* = 4 independent experiments). Microtubule cell–cell interconnection markedly disarranged upon cisplatin exposure in parallel to mitochondria re-distribution in the perinuclear region of injured RPTECs. Co-culture with UC-MSCs normalized RPTEC tubulin cytoskeletal architecture and the mitochondrial distribution along tubulin-rich protrusions. Nuclei are counterstained with DAPI (blue). Scale bar 10 μm. Data in **b** are expressed as mean ± SEM

Horizontal trafficking of functional mitochondria among cells has recently emerged as a new physiological and rescuing process in a plethora of different stress conditions^33–40^. We therefore explored whether UC-MSCs could induce mitochondrial transfer among injured RPTECs through cytoplasmic protrusions. First, we observed that the development of tubular cell cytoplasmic projections normally occurred in control cells (Fig. 4a) and strongly declined after cisplatin exposure (Fig. 4a). Co-culture with UC-MSCs markedly enhanced cell–cell connections among cisplatin-injured RPTECs (Fig. 4a). Then, to demonstrate the intercellular mitochondria transport among RPTECs, we used two fusion constructs of the E1 alpha pyruvate dehydrogenase leader sequence with GFP or RFP, which efficiently labeled tubular cell mitochondria in green and red, respectively. Two distinct populations of RPTECs were obtained by separately transfecting cells with GFP- or RFP-tagged mitochondria. Cells were then mixed and seeded in equal proportions. The physiological exchange of mitochondria occurred among RPTECs, to the extent that 18% of resting cells showed both red and green (yellow) mitochondria (Fig. 4b). Exposure to cisplatin reduced the organelle transfer to only 4.1% of the cells, which was instead restored to 25% by co-culture with UC-MSCs, as evidenced by the presence of yellow mitochondria within RPTEC cellular projections (Fig. 4b). Incubation of injured RPTECs with a non-toxic concentration of taxol (see Methods section), a suppressor of microtubule dynamics, prevented the formation of cytoplasmic protrusions (Supplementary Fig. 6B) and the mitochondrial trafficking (Fig. 4b) induced by UC-MSCs, suggesting that tubulin is directly involved in both processes.

This evidence was supported by data of co-staining of tubulin and SOD2-labeled mitochondria that disclosed a physiologically microtubule interconnection with mitochondria among resting RPTECs (Fig. 4c). Following cisplatin treatment, tubulin cytoskeleton architecture disarranged and mitochondria distributed in clusters in the perinuclear region (Fig. 4c). Co-culture with UC-MSCs normalized the microtubule network and restored mitochondrial distribution within cell–cell projections (Fig. 4c).

### UC-MSCs regulate mitochondrial trafficking via Kif5c

**Fig. 5:**
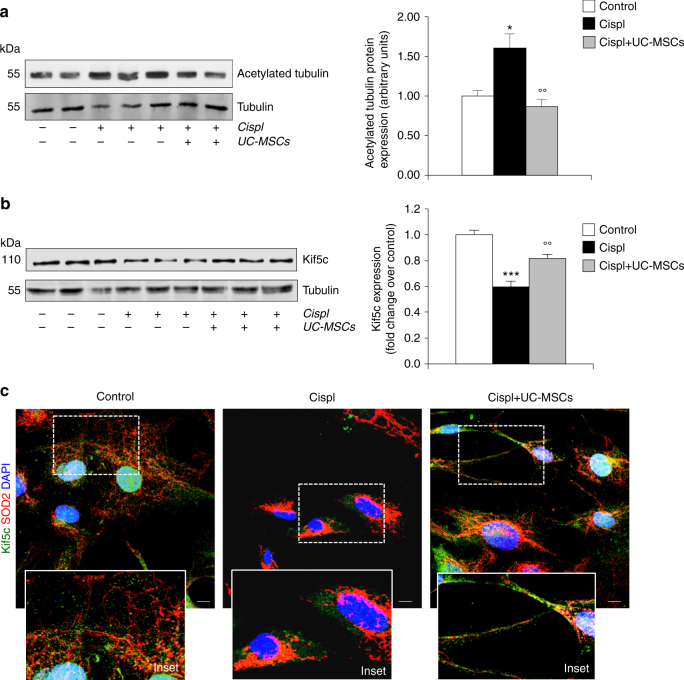
Human UC-MSCs enhance mitochondrial trafficking through microtubule preservation and Kif5c expression in injured tubular cells. **a** Representative western blots and densitometric analysis of acetylated tubulin in control RPTECs (*n* = 5), cisplatin-treated RPTECs alone (*n* = 6), or co-cultured with UC-MSCs (*n* = 6). *P < 0.05 vs Control and °°*P* < 0.01 vs Cispl using ANOVA corrected with Bonferroni coefficient. **b** Representative western blots and densitometric analysis of Kif5c in control RPTECs, cisplatin-treated RPTECs alone or co-cultured with UC-MSCs (*n* = 6 independent experiments). ***P < 0.001 vs Control and °°*P* < 0.01 vs Cispl using ANOVA corrected with Bonferroni coefficient. **c** Representative images of immunofluorescence analysis of Kif5c (green) and SOD2-labeled mitochondria (red) in control RPTECs, cisplatin-treated RPTECs alone, or co-cultured with UC-MSCs (*n* = 3 independent experiments). Nuclei are counterstained with DAPI (blue). Enlarged details of preserved Kif5c that co-localized with SOD2-labeled mitochondria in cisplatin-treated RPTECs exposed to UC-MSCs in respect to RPTECs treated with cisplatin alone, are shown in the insets. Scale bar 10 μm. Data in **a** and **b** are expressed as mean ± SEM

We next investigated the mechanism underlying the effect of human UC-MSCs in favouring the formation of intercellular processes—a condition indispensable for mitochondrial trafficking—by assessing whether human UC-MSCs were able to regulate post-translational modifications of microtubules in cultured RPTECs. As shown in Fig. 5a, cisplatin induced a shift toward the acetylated form of tubulin compared to control cells. Treatment with UC-MSCs restored the deacetylation levels of tubulin in cisplatin-injured RPTECs (Fig. 5a) possibly by regulating SIRT3 activity.

It is known that the motor protein Kif5c, a kinesin heavy chain subunit, is involved in the anterograde transport of mitochondria along the microtubule network^41, 42^. Tubular cells constitutively express Kif5c, which decreased in response to cisplatin (Fig. 5b). UC-MSC treatment enhanced tubular expression of Kif5c (Fig. 5b). Consistently, immunofluorescence analysis showed that Kif5c appeared as punctuate cytoplasmic staining in RPTECs under physiological conditions (Supplementary Fig. 6C). When RPTECs were exposed to cisplatin, a marked reduction in Kif5c was observed, which was increased by UC-MSC co-incubation (Supplementary Fig. 6C). That Kif5c actually interacts with mitochondria was shown in resting RPTECs by co-staining with SOD2-labeled mitochondria (Fig. 5c). At variance, cisplatin induced a strong perinuclear localization of mitochondria and Kif5c (Fig. 5c). A close connection between Kif5c and mitochondria was found along the tubulin-rich projections in injured RPTECs in response to UC-MSCs (Fig. 5c).

The finding that F-actin did not co-stain with mitochondria (Supplementary Fig. 6D), ruled out the possibility that F-actin plays a role in mitochondrial transport.

### SIRT3 preserves mitochondria and their exchange among cells

**Fig. 6:**
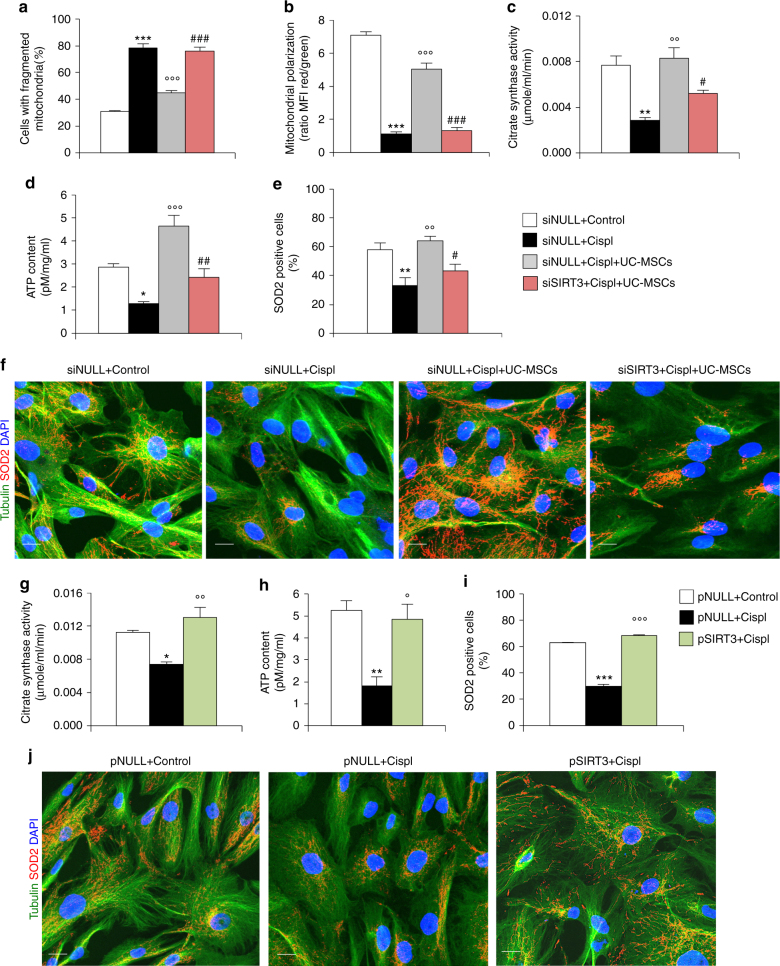
SIRT3 regulates mitochondrial integrity and tubulin-dependent exchange in injured RPTECs. **a** Quantification of mitochondrial fragmentation assessed by MitoTracker staining and **b** quantification (red/green florescent area) of mitochondrial membrane potential by JC-1 in RPTECs transfected with null small interfering RNA (siNULL) or small interfering for SIRT3 (siSIRT3) and incubated with cisplatin alone or co-cultured with UC-MSCs (*n* = 4 independent experiments). Control RPTECs transfected with siNULL were used for comparison. ***P < 0.001 vs siNULL + Control, °°°*P* < 0.001 vs siNULL + Cispl, and ###*P* < 0.001 vs siNULL + Cispl + UC-MSCs using ANOVA corrected with Bonferroni coefficient. **c** Evaluation of citrate synthase activity, **d** ATP content, and **e** SOD2 protein expression in RPTECs transfected with siNULL or siSIRT3, and then treated with cisplatin alone or co-cultured with UC-MSCs (*n* = 3 independent experiments). Control RPTECs transfected with siNULL were used for comparison. *P < 0.05 and **P < 0.01 vs siNULL + Control, °°*P* < 0.01 and °°°*P* < 0.001 vs siNULL + Cispl, #*P* < 0.05 and ##*P* < 0.01 vs siNULL + Cispl + UC-MSCs using ANOVA corrected with Bonferroni coefficient. **f** Representative images showing that UC-MSCs were incapable to restore tubulin network (green) and SOD2-labeled mitochondria (red) distribution when co-cultured with cisplatin-treated RPTECs silenced for SIRT3 (siSIRT3) in respect to cells transfected with siNULL (*n* = 3 independent experiments). Control RPTECs transfected with siNULL were used for comparison. Nuclei are counterstained with DAPI (blue). Scale bar 10 μm. **g** Evaluation of citrate synthase activity, **h** ATP content, and **i** SOD2 protein expression in RPTECs overexpressing SIRT3 by lipofectamine-mediated transfection with pCMV-hSIRT3-GFP (pSIRT3) in comparison with pCMV6-AC-GFP (pNULL) and then exposed to control medium or cisplatin (*n* = 4 independent experiments). *P < 0.05, **P < 0.01, and ***P < 0.001 vs pNULL + Control; °*P* < 0.05, °°*P* < 0.01, and °°°*P* < 0.001 vs pNULL + Cispl using ANOVA corrected with Bonferroni coefficient. **j** Representative images of immunofluorescence analysis showing that SIRT3 overexpression in RPTECs transfected with pSIRT3 preserved tubulin network (green) and SOD2-labeled mitochondria (red) re-distribution in response to cisplatin as compared to cisplatin-treated cells transfected with pNULL (*n* = 4 independent experiments). Nuclei are counterstained with DAPI (blue). Scale bar 10 μm. Data in **a**–**e** and **g**–**i** are expressed as mean ± SEM

Having established a strong correlation between the renoprotective effect of UC-MSCs and restoration of SIRT3 expression and activity, we investigated in vitro whether SIRT3 could be a MSC target actually not dispensable in preserving mitochondrial integrity and favouring tubular cell repair. For this purpose, SIRT3 was silenced in cultured RPTECs by specific small interfering RNA (siRNA) to obtain a remarkable reduction in SIRT3 mRNA expression as compared to cell transfected with siNULL (siSIRT3: 0.4 ± 0.04 vs siNULL: 1.1 ± 0.01, *P* < 0.01 with unpaired *t*-test, *n* = 3 independent experiments). Notably, UC-MSCs co-cultured with RPTECs silenced for SIRT3 failed to normalize cisplatin-induced mitochondrial fragmentation (Fig. 6a) and depolarization (Fig. 6b). Concomitantly, UC-MSCs were unable to recover mitochondrial mass (Fig. 6c) and ATP levels (Fig. 6d) in damaged RPTECs lacking SIRT3. Moreover, the ability of UC-MSCs to enhance SOD2 expression (Fig. 6e) and mitochondrial shuttling among injured tubular cells through tubulin-rich protrusions (Fig. 6f) was abolished by SIRT3 silencing in cisplatin-injured RPTECs.

A further proof of the functional role of SIRT3 in tubular cell repair was documented in SIRT3 overexpressing RPTECs, a condition that mimics UC-MSC effect. Upregulation of SIRT3 mRNA in cultured RPTECs was obtained by lipofectamine-mediated transfection with pCMV-hSIRT3-GFP (pSIRT3) as compared to cells transfected with pCMV6-AC-GFP (pNULL) (pSIRT3: 2.3 ± 0.13 vs pNULL: 1.0 ± 0.03, *P* < 0.001 with unpaired *t*-test, *n* = 3 independent experiments). SIRT3 overexpression protected RPTECs from cisplatin-induced mitochondrial fragmentation and depolarization, as previously documented by our group^20^. Likewise, here we found that cisplatin did not reduce mitochondrial mass (Fig. 6g) and ATP production (Fig. 6h) in RPTECs in face of high SIRT3 expression as compared to cells transfected with pNULL. We also found that SOD2 expression was not affected by cisplatin exposure in tubular cells overexpressing SIRT3 as compared to cells transfected with pNULL (Fig. 6i). In line with previous findings showing that microtubule distribution and function are under the control of NAD^+^ and SIRT3 deacetylase activity^43^, here we found that the formation of cytoplasmic protrusions rich in mitochondria was preserved in injured RPTECs expressing high levels of SIRT3 (Fig. 6j). Collectively, all these data clearly indicate SIRT3 as a crucial player in the process of tubular repair induced by UC-MSCs.

### UC-MSCs drive tubular repair regulating SIRT3 targets in AKI

**Fig. 7:**
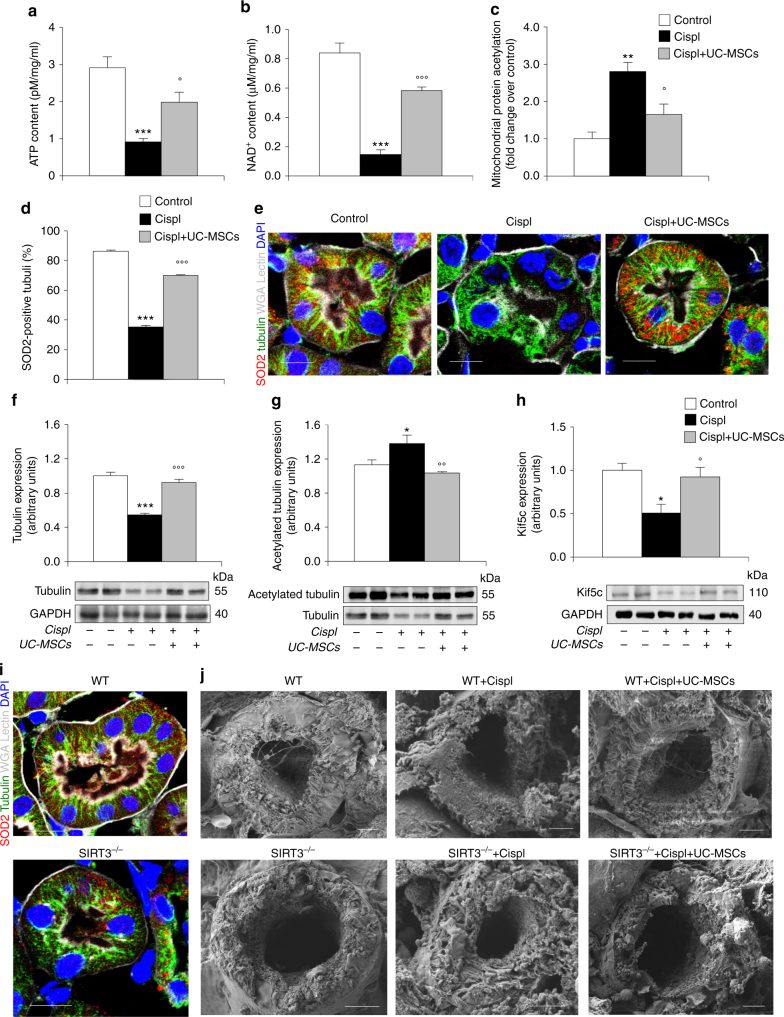
Human UC-MSC treatment preserves mitochondrial function and tubulin-dependent exchange by regulating SIRT3 targets in renal tissue of AKI mice. **a** Evaluation of ATP and **b** NAD^+^ content in renal tissue from control and cisplatin mice (NOD/SCID) given saline or UC-MSCs at 4 days (*n* = 4 mice per group). ***P < 0.001 vs Control and °*P* < 0.05, °°°*P* < 0.001 vs Cispl using ANOVA corrected with Bonferroni coefficient. **c** Densitometric analysis of protein acetylation in mitochondria isolated from renal tissue of control and cisplatin mice given saline or UC-MSCs at 4 days (*n* = 3 mice per group). **P < 0.01 vs Control and °*P* < 0.05 vs Cispl using ANOVA corrected with Bonferroni coefficient. **d** Quantification of SOD2 protein expression (red) in proximal tubuli of control and cisplatin mice given saline or UC-MSCs at 4 days (*n* = 6 mice per group). ***P < 0.001 vs Control and °°°*P* < 0.001 vs Cispl using ANOVA corrected with Bonferroni coefficient. **e** Representative images showing that in proximal tubuli of cisplatin mice at 4 days, the expression of SOD2 (red) and tubulin (green) was decreased and their distribution was markedly altered in respect to control animals; both proteins were normalized by UC-MSCs. The tubuli are labeled with wheat germ agglutinin (WGA) lectin (white), and nuclei with DAPI (blue). Scale bar 10 μm. **f** Representative western blots and densitometric analysis of tubulin, **g** acetylated tubulin, and **h** Kif5c protein expression in renal tissue of control and cisplatin mice given saline or UC-MSCs at 4 days (*n* = 4 mice per group). *P < 0.05, ***P < 0.001 vs Control and °*P* < 0.05, °°*P* < 0.01, °°°*P* < 0.001 vs Cispl using ANOVA corrected with Bonferroni coefficient. **i** Representative immunofluorescence images of SOD2 (red) and tubulin expression (green) in proximal tubuli of untreated wild-type (WT) littermates that markedly changed in SIRT3-deficient (SIRT3^−/−^) mice. Tubuli are identified with WGA lectin (white) and nuclei with DAPI (blue). Scale bar 10 μm. **j** Scanning electron microscopy micrographs showing the lumen of proximal tubuli of untreated WT littermates and SIRT3^−/−^ mice. No nanotube-like structures were observed in untreated SIRT3^−/−^ mice. Cisplatin affected nanotube-like structures in tubuli of WT mice that were restored by UC-MSCs at variance with SIRT3^−/−^ cisplatin mice that did not respond to UC-MSCs. Scale bar 4 μm. Data in **a**–**d** and **f**–**h** are expressed as mean ± SEM

The biological relevance of our in vitro findings was validated in mice with cisplatin-induced AKI. First, we found that ATP levels were markedly lower in kidney extracts of cisplatin-injected mice compared to control animals, and were enhanced following UC-MSC treatment (Fig. 7a). Concomitantly, the mRNA expression of ATP synthase, the oxidative phosphorylation complex V responsible for the ATP production, was recovered by UC-MSCs in renal tissues of cisplatin mice (Supplementary Fig. 7A). We also found a reduction in NAD^+^ content in renal tissues harvested from mice with cisplatin-induced AKI, which was restored in mice treated with UC-MSCs (Fig. 7b). NAD^+^ replenishment was accompanied by a normalization of the acetylome profile of mitochondrial proteins isolated from the renal tissue of cisplatin mice infused with UC-MSCs compared to mice given saline (Fig. 7c; Supplementary Fig. 7B), thus reflecting a boost in SIRT3 deacetylase activity. In the renal tissues of cisplatin mice, SIRT3 transcript level (Supplementary Fig. 7C) and protein (Supplementary Fig. 7D) decreased compared to control animals, and was markedly restored by UC-MSCs treatment. A similar trend was observed for the expression of the upstream regulator of SIRT3, the transcription factor PGC1α, that markedly decreased in renal tissue of cisplatin mice and was upregulated by UC-MSC treatment (Supplementary Fig. 7E). In parallel, SOD2 protein expression recovered in response to UC-MSC treatment (Fig. 7d, e; Supplementary Fig. 7F, G).

Consistent with in vitro data, changes and rarefaction of microtubule distribution pattern in proximal tubuli of mice with cisplatin-induced AKI (Fig. 7e; Supplementary Fig. 7G) were associated with total tubulin downregulation (Fig. 7f) and increased acetylated tubulin expression (Fig. 7g). In cisplatin mice given UC-MSCs, microtubule distribution normalized (Fig. 7e; Supplementary Fig. 7G) concomitantly with the restoration of tubulin protein expression (Fig. 7f) and its acetylated status (Fig. 7g). Moreover, the expression of the motor protein Kif5c, which markedly decreased in response to cisplatin, was restored to normal levels following UC-MSC treatment (Fig. 7h).

As proof-of-concept that SIRT3 has a role in the regulation of microtubule architecture, we took advantage of untreated SIRT3-deficient mice that, compared to their untreated wild-type (WT) littermates, showed a constitutive hyper-acetylation state of mitochondrial proteins (Supplementary Fig. 8A), an altered distribution and reduced expression of SOD2 and tubulin (Fig. 7i; Supplementary Fig. 8B), similar to that observed in mice with cisplatin-induced AKI showed in Fig. 7e. Scanning electron microscopy (SEM) of renal tissue from untreated WT mice revealed the presence of several thin cytoplasmic protrusions, with nanotube-like morphology, which arose from the apical part of the tubular cells and crossed the lumen connecting different epithelial cells from the same proximal tubule (Fig. 7j). At variance, untreated mice deficient in SIRT3 did not show any signs of cell protrusion in the lumen of proximal tubuli (Fig. 7j). In these animals lacking SIRT3, cisplatin injection induced a more severe renal dysfunction and tubular injury (Supplementary Fig. 8C, D) as compared with those observed in WT littermates with AKI (Supplementary Fig. 8E, F). Notably, lack of any protective effect on renal BUN levels, tubular damage (Supplementary Fig. 8C, D) and nanotube formation (Fig. 7j) was observed when cisplatin SIRT3^−/−^ mice were infused with UC-MSCs. At variance, in the WT littermates with AKI, infusion of UC-MSCs or their corresponding conditioned medium (CM) (Supplementary Fig. 8E, F) markedly protected from renal functional and structural impairment. In parallel, nanotube-like structures, which were affected by cisplatin injection in WT mice, were restored by UC-MSCs (Fig. 7j). Altogether these data clearly demonstrate a key role of SIRT3 in the process of renal repair in AKI.

## Discussion

In this study, we provide direct experimental evidence that human UC-MSC therapy facilitates tubular cell recovery, thus preserving mitochondrial functional integrity and mobility as well as restoring the microtubule network, which are pivotal determinants of bioenergetic crosstalk among injured cells. Here, we found that after systemic infusion in mice with AKI, UC-MSCs localized in the damaged kidney almost exclusively in peritubular areas and strongly limited cisplatin-induced tubular cell mitochondrial fragmentation, thus tipping mitochondrial dynamics toward fusion as opposed to fission. Tubular cell recovery was specifically induced by human UC-MSCs and by their CM through a paracrine action on resident renal cells, at variance of human fibroblasts, here used as control cells, which failed to improve mitochondrial damage and renal injury in cisplatin mice. These findings were corroborated by in vitro studies, which established a strong causal link between the protective paracrine effect of UC-MSCs and the preservation of mitochondrial functional, and structural integrity in cisplatin-injured cultured tubular cells.

To gain a better understanding of the mechanisms underlying the beneficial effect of MSC therapy, we first performed a genome-wide differential gene expression analysis of cultured tubular cells. Notably, we found a remarkable difference in gene expression profile between resting and injured tubular cells, especially when exposed to UC-MSCs, suggesting that cell-based therapy primes the transcriptional induction of several genes in tubular cells that are possibly involved in the processes of renal repair. Notably, enrichment analysis revealed a stark difference between resting cells and injured RPTECs exposed to UC-MSCs, in terms of a high recurrence of genes involved in mitochondrial energy production, including aminoacid catabolism, urea cycle, fatty acid metabolism, and electron transport chain components. We also found that AMPK—a metabolic sensor that maintains energy homeostasis—was upregulated by UC-MSCs, in line with a previous finding of ours showing that AMPK activation by AICAR exerted a renoprotective effect^20^. Concurrently, *Nampt*—an AMPK downstream target^44^—and kynureninase were strongly upregulated by UC-MSCs, both regulators of their biosynthetic product NAD^+^, which we found actually increased at the tubular cell intracellular level in response to MSCs compared to cisplatin-injured cells. Collectively, all these data strongly indicated that UC-MSCs were able to induce global metabolic reprogramming of damaged tubular cells to sustain energy supply. Consistently, it has recently been demonstrated that NAD^+^ plays a role in rewiring tubular cell metabolism to sustain renal recovery in different experimental models of AKI^45^. NAD^+^ is the essential cofactor of several enzymes, including the deacetylase activity of SIRT3^46^, which controls the mitochondrial adaptive stress response^47, 48^ and energy production^21^. The biological relevance of SIRT3 as an essential player in tubular cell repair in experimental AKI was supported by the finding that renal SIRT3 reduction was functionally linked to a striking impairment of mitochondrial dynamics^20^. The above evidence offered the rationale for looking at SIRT3 as a possible key driver of UC-MSC regenerative activity in tubular cells during AKI. Notably, we found that UC-MSCs preserved both SIRT3 deacetylase activity and expression, enhancing tubular ATP and SOD2 levels, thereby preventing the energy deficit and sustaining the antioxidant activity. The mechanism by which MSC modulate mitochondrial SIRT3 expression could be explained by the ability of UC-MSCs to regulate the intracellular level of Nampt and PGC1α, which are known to induce SIRT3 gene transcription^25^. The evidence of a direct role of SIRT3 in the process of tubular cell repair was provided by transfection experiments in which SIRT3 overexpression in RPTECs prevented the mitochondrial dysfunction triggered by cisplatin, while the lack of SIRT3 in injured tubular cells was enough to abolish the protective effect of UC-MSCs on mitochondrial functional integrity. The translational relevance of these in vitro findings rests on data that SIRT3-deficient mice exhibited a more severe AKI compared to their WT littermates, and did not respond to UC-MSC treatment, thus underlying the essential contribution of SIRT3, a key target of cell therapy, in the process of tubular repair. We can not definitely rule out the theoretical possibility that substance(s) secreted by UC-MSCs may protect from cisplatin-induced injury by lowering some critical contributors to cell death independently of SIRT3. This however is inconsistent with evidence provided here that UC-MSCs failed to protect both SIRT3^−/−^ mice and SIRT3-deficient RPTECs from cisplatin-induced injury.

Intercellular mitochondrial transfer from donor cells to injured tissues has recently been described as a new rescue mechanism that counteracts a plethora of different stress conditions^33–40^. Here, the relevance of mitochondrial cell–cell trafficking has been highlighted by findings showing that RPTEC constitutively exhibited a complex interconnected microtubule network exchanging a large number of mitochondria via the motor protein Kif5c. This mechanism was markedly affected in cisplatin-injured tubular cells. Treatment with UC-MSCs strongly enhanced mitochondrial intercellular transport along the tubulin-rich protrusions, a phenomenon that was halted by taxol, indicating that microtubules are active players in organelle mobility. These long-distance cell–cell connections, recently defined as membrane nanotubes^49^, are sites of mitochondrial transfer, a process that bridges energy exchange to complement cellular metabolism^37, 50–53^. These data advance our understanding of the complex pro-regenerative paracrine effects of UC-MSCs and disclose that, at least in the kidney, UC-MSCs may not act directly like mitochondrial donors, as previously stated^34–36, 54, 55^ and here documented by the lack of human mitochondria (hMT) within mouse tubular cells in proximity of engrafted MSCs. Instead, UC-MSC therapy regulates and protects the molecular machinery that drives the intercellular transfer of healthy mitochondria among tubular cells during injury, favouring bioenergetic crosstalk.

Having established that UC-MSCs play an important role in both mitochondrial motility and SIRT3 expression and activity, we further investigated whether SIRT3 could be the driving force in microtubule-dependent mitochondrial transfer. A recent study pointed to SIRT3 being a regulator of microtubule dynamics in terms of tubulin polymerization and depolimerization^43^. Furthermore, the deacetylation state of tubulin has been described as affecting microtubule intracellular dislocation^56, 57^ and function in dynamic cellular projections^58^. Our data showed that treatment with UC-MSCs normalized the distribution and acetylation of tubulin in injured tubular cells and in mice with AKI. That SIRT3 plays a functional role in regulating mitochondrial spatial distribution along intercellular tubulin-rich projections was further supported by the evidence that SIRT3 overexpression in tubular cells prevented the cisplatin-induced reduction of cytoplasmic protrusions carrying mitochondria, while SIRT3 silencing strongly halted the ability of UC-MSCs to induce mitochondrial trafficking among RPTECs. The ability of SIRT3 to regulate mitochondrial motility can be attributed to its capacity to control ROS generation^23^ and AMPK activation^59^, which have been shown to regulate tubulin acetylation^60–62^ and cellular mitochondrial flux^63^, respectively. Finally, taking advantage of genetically modified mice, we demonstrated that SIRT3 deficiency caused a strong reduction in tubulin expression in proximal tubular cells, a condition observed in WT mice with AKI that exhibit SIRT3 downregulation. The observed microtubule disassembly in SIRT3^−/−^ mice translated into a complete lack of nanotube-like structures among tubular cells, clearly indicating that SIRT3 modulated the microtubule network.

In conclusion, we uncovered that human UC-MSCs carry out a multimodal paracrine action in which complex intra- and inter-cellular pathways promote reparative mechanisms in experimental AKI by preserving mitochondrial homeostasis and microtubule-dependent organelle trafficking through the enhancement of SIRT3 activity. These data highlight the potential that therapies with MSCs or their bio-products have as future translational approaches in humans. In addition to the clinical implications for AKI, these data provide new clues regarding how the endosymbiotic origins of mitochondria could be exploited by cell-based therapy to promote intercellular trafficking, driving the high-energy process of repair in organs other than the kidney.

## Methods

### In vivo experimental design

Two-month-old immunodeficient NOD/SCID female mice (Charles River Italia S.p.a., Calco, Italy) were randomly allocated to the experimental groups. Animals were housed in a constant room temperature (22–24 °C) with a 12-hour dark and 12-hour light cycle and fed a standard diet. AKI was induced in NOD/SCID mice by subcutaneous injection of the nephrotoxic drug *cis*-diaminedichloroplatinum (cisplatin; Ebewe Italia Srl, Rome, Italy) at the dose of 16.0 mg/kg. To investigate the effect of human UC-derived MSC (UC-MSC) treatment in AKI, mice were divided into two groups and intravenously (i.v.) injected 24 h after cisplatin as follows: group 1, saline (*n* = 6); group 2, UC-MSCs at sixth passage (5 × 10^5^ cells per mouse; *n* = 6). Normal mice served as controls (*n* = 6). Mice were killed at 4 days after cisplatin and kidney samples were collected. Renal function was assessed as BUN using the Reflotron test (Roche Diagnostics, Milan, Italy) following manufacturer’s instructions. The effect of i.v. infusion of human fibroblasts at sixth passage (5 × 10^5^ cells per mouse; *n* = 4), the same passage of UC-MSCs, has been assessed in NOD/SCID mice 24 h after cisplatin injection, in comparison to saline mice (*n* = 4), and killed at 4 days to assess renal function, histology, and mitochondrial morphology.

In selected experiments, 2-month-old SIRT3^−/−^ female mice, generated in a mixed genetic background (provided by Professor Frederick Alt, Harvard Medical School, Boston, MA, USA)^32^, and their C57BL6x129 WT littermates were randomly allocated to the experimental groups as follows: group 1, SIRT3^−/−^ mice received saline (*n* = 4); group 2, SIRT3^−/−^ mice received cisplatin and saline (*n* = 6); group 3, SIRT3^−/−^ received cisplatin and, after 24 h, UC-MSCs (5 × 10^5^ cells per mouse, *n* = 6); group 4, C57BL6x129 mice received saline (*n* = 5); group 5, C57BL6x129 mice received cisplatin and saline (*n* = 5); group 6, C57BL6x129 mice received cisplatin and, 24 h later, UC-MSCs (5 × 10^5^ cells per mouse, *n* = 6); group 7, C57BL6x129 mice received cisplatin and, 24 h later, CM derived UC-MSCs (CM derived from 5 × 10^5^ UC-MSCs/mouse, *n* = 5). All these animals were killed at 4 days to assess renal function, histology, and SEM analysis.

### Renal histology

Kidney samples were fixed in Duboscq-Brazil, and paraffin sections were stained with periodic acid—Schiff’s reagent (PAS). Luminal hyaline casts and tubular necrosis (denudation of the tubular basement membrane) were assessed in non-overlapping fields (up to 28 for each section) using a ×40 objective (high-power field, HPF; Primo Star, Zeiss, Jena, Germany). The number of casts and tubular profiles showing necrosis was analyzed twice in a single-blind fashion.

### Morphometric analysis of mitochondria in tubular cells

Glutaraldehyde-fixed fragments of cortical kidney tissue were washed repeatedly in cacodylate buffer, postfixed in 1% OsO_4_, dehydrated through ascending grades of alcohol, and embedded in Epon resin. Ultrathin sections were stained with UAR-uranyl acetate replacement stain for examination using a Philips Morgagni transmission electron microscope (TEM; Philips). Numerical density of mitochondria (*N*_V_, n/µm^3^) was estimated using morphometric analysis according to Weibel^64^, using an orthogonal grid digitally superimposed to digitized electron microscope pictures of proximal tubules at ×7100. Briefly, the mitochondrial profile area density (*N*_A_) was estimated using the ratio between the number of mitochondria and the proximal tubular area in the image calculated on the basis of grid points. Mitochondrial volume density (*V*_V_) was determined using the ratio of grid points falling over mitochondria, divided by the total number of points of the grid container in the proximal tubule section. *N*_V_ was then estimated for each animal using the formula^64^:

$$N_{\rm{V}} = \left( {1/{\beta}} \right)\left( {N_{\rm{A}}^{3/2}/V_{\rm{V}}^{1/2}} \right)$$

Where *β* is the shape coefficient for ellipsoidal mitochondria, calculated as the ratio of the harmonic mean of major and minor axis of mitochondrial sections measured on digital images. The mean mitochondrial volume was calculated for each animal as the ratio of mitochondrial volume density *V*_V_ to numerical density *N*_V_.

### Scanning electron microscopy

Kidneys of SIRT3^−/−^ mice and their WT littermates perfused with glutaraldehyde 1.25% were analyzed for SEM. Renal specimens were fixed in 2.5% glutaraldehyde, washed in cacodylate buffer and postfixed in 1% osmium tetroxide. Samples were then dehydrated in graded ethanol, critical point-dried in carbon dioxide and coated with atomic gold particles prior to viewing with a SEM (Supra 55, Zeiss, Oberkochen, Germany)^65^.

### Immunofluorescence analysis in renal tissue

For SOD2 staining in renal tissue, 3 µm optimal cutting temperature (OCT)-fixed cryosections were air-dried. After blocking non-specific sites with 1% bovine serum albumin (BSA, Sigma-Aldrich, Milan, Italy) in phosphate-buffered saline (PBS), slides were incubated with rabbit anti- SOD2 (1:200; #AB10346; Merck Millipore, Darmstadt, Germany) antibody, followed by donkey anti-rabbit Cy3-conjugated secondary antibodies (1:80; Jackson Immunoresearch Laboratories, West Grove, PA, USA). For tubulin or SIRT3 staining in renal tissue, 3 µm periodate-lysine paraformaldehyde (PLP)-fixed cryosections were air-dried and washed with PBS. Then samples were incubated with 3% BSA, 10% fetal calf serum (FCS) in PBS to block non-specific sites for 30 min, followed by a mouse anti-tubulin (1:100; #T9026 clone DM1A; Sigma-Aldrich) or a rabbit anti-SIRT3 antibody (1:50, #sc-49744 clone P-19; Santa Cruz) overnight at 4 °C, followed by a donkey anti-mouse or anti-rabbit FITC-conjugated (1:80; Jackson Immunoresearch Laboratories) antibody, as appropriate. Nuclei were stained with 4′,6-diamidino-2-phenylindole (DAPI, Sigma-Aldrich) and the renal structure was labeled with FITC-conjugated wheat germ agglutinin (WGA) lectin (1:500; #FL-1021S; Vector Laboratories, Burlingame, CA, USA), rhodamine-labeled WGA lectin (1:500; #RL-1022; Vector Laboratories), or Alexa Fluor 633-conjugated WGA (1:100; #W21404; Thermo Fisher, Life Technologies; Waltham, MA, USA), as appropriate. Samples were examined by confocal inverted laser microscopy (LSM 510 Meta, Zeiss) and the quantification of SOD2 was performed in 10 random fields per sample and expressed as the % of SOD2-positive tubuli and normalized for the total number of tubuli.

### Quantification of UC-MSCs engraftment

To study the engraftment of human UC-MSCs in mouse renal tissues, HNA was used. Briefly, 8 μm thick sections from OCT-included kidney specimens were fixed 10 min in cold acetone at 4 °C. Then, sections were incubated with 1% BSA blocking solution in PBS and then incubated with anti-human Nuclei-Fluor 488-conjugated antibody (1:50; #MAB1281A4 clone 235-1; Merck Millipore) at 4 °C overnight. In addition, a mouse anti-human mitochondria (hMT) Cy3-conjugated antibody (1:50; #MAB1273C3 clone 113-1; Merck Millipore) was used. Nuclei were counterstained with DAPI and the renal structure was marked with Alexa Fluor 633-Conjugated WGA (1:100; #W21404; Thermo Fisher, Life Technologies) for 10 min Cover slips were mounted using fluorescent mounting medium (Dako, Agilent Technologies, Santa Clara, CA, USA). Twelve sections for each animal (*n* = 5) were analyzed at confocal microscope (Zeiss, Original magnification ×630), and HNA-positive cells were counted. Data were expressed as the number of HNA-positive cells per 1 × 10^5^ renal cells.

### Real-time PCR

Renal tissue was harvested in TRIzol reagent (Thermo Fisher, Life Technologies) and total RNA was extracted according to the manufacturer’s instructions. Contaminating genomic DNA was removed by RNase-free DNase (Promega, Madison, WI, USA) for 1 h at 37 °C. The first-strand complimentary DNA (cDNA) (2 µg) was produced using the SuperScript II cDNA Synthesis Kit (Thermo Fisher, Life Technologies) following the manufacturer’s procedure. No enzyme was added for reverse transcriptase-negative controls (RT−).

To amplify cDNA of mouse *Atp5b, Pgc1α*, and *Sirt3*, we used TaqMan Universal PCR Master Mix (Applied Biosystems) and inventoried TaqMan assays of the *Atp5b* (FAM/MBG probe Mm00443967g1), the *Pgc1α* gene (FAM/MBG probe Mm00447181m1), the *Sirt3* gene (FAM/MBG probe Mm01275638m1), and a mouse α-actin endogenous control (VIC/MGB probe), according to the manufacturer’s instructions. PCR was performed on the Viia7 Real-Time PCR System (Applied Biosystems). The amplification profile consisted of 2 min at 50 °C and 10 min at 95 °C; the samples were cycled 40 times at 95 °C for 15 s. and 60 °C for 60 s. Data were analyzed using the 2^−ΔΔCT^ method and presented as fold changes relative to mouse wild type (Control).

To amplify cDNA of human SIRT3 and GAPDH, inventoried TaqMan assays (FAM/MBG probe Hs_00202030m1 and 4333764F, respectively) were used according to the manufacturer’s instructions.

### Cell cultures and incubations

Human UC-derived MSCs (UC-MSCs) were collected from pregnant women after cesarean sections and informed written consent was obtained. Samples were maintained at 4 °C and then processed in accordance with a good manufacturing practice (GMP)-compliant protocol for the isolation and expansion of UC-MSCs^66^. Briefly, after rinsing the vein by blunt end needle syringe, the UC was cut in 5 cm long segments and subsequently cut longitudinally to expose the inner surface and subsequently transferred to a 150 mm Petri dish (Corning, New York, NY, USA) containing alpha MEM (Thermo Fisher, Invitrogen) enriched with 5% human platelet lysate (hPL) from healthy donors^67^ and gentamicin and kept at 37 °C in 5% CO_2_. After 1 week, UC remaining tissue was removed and adherent cells expanded for an additional week, changing 40% medium every 3–4 days. At sub confluency, cells were detached by TrypLe (Thermo Fisher) treatment and reseeded for two consecutive expansion steps at 100–300 cells/cm^2^, until confluency, in CellSTACK (Corning) chambers^66^.

Immunophenotypic analysis revealed that UC-MSCs showed a strong expression of the main MSC markers CD73, CD90, and CD105, as well as of CD44 and CD166. Additionally, UC-MSCs possessed a high proliferative capacity, as evidenced by colony forming unit (CFU-F) assays^66^. When tested for differentiation capabilities, UC-MSCs were able to differentiate into adipogenic, chondrogenic, and osteogenic lineages, thus indicating that UC-MSCs are multipotent stromal cells^66^.

For the experiments, human UC-MSCs were grown in culture medium, consisting of alpha MEM (Invitrogen) enriched with 5% hPL and 50 mg/ml gentamicin in a humidified atmosphere of 5% CO_2_ at 37 °C. At 80% confluence, cells were detached and seeded (10,000 cells/cm^2^) up to a maximum eighth passage.

As control cells, adult normal human dermal fibroblasts (NHDF-Ad) were purchased from Lonza (#CC-2511) and grown in fibroblast basal medium (FBM; Lonza, Milan, Italy) supplemented with FGM-2 single quotes (Lonza). NHDF-Ad are guaranteed to test negative for mycoplasma, bacteria, yeast, and fungi. NHDF-Ad are characterized by morphological observation throughout serial passages. A certificate of analysis is provided by Lonza for each cell lot.

Human RPTECs were purchased from Lonza (#CC-2553) and grown in renal epithelial cell basal medium (REBM; Lonza) supplemented with REGM SingleQuots (Lonza) in a humidified atmosphere of 5% CO_2_ at 37 °C. At 80% confluence, RPTECs were detached by trypsinization and reseeded (5000 cells/cm^2^) up to a maximum 10th passage. RPTECs are guaranteed to test negative for mycoplasma, bacteria, yeast, and fungi. RPTEC are characterized by positivity for gamma-glutamyl transpeptidase (γ-GTP) and alkaline phosphatase, and by their ability to form tubules on Matrigel. A certificate of analysis is provided by Lonza for each cell lot.

For all experiments, RPTECs were seeded 30,000 cells/cm^2^ and exposed to cisplatin 5 μM for 6 h (in REBM basal medium, Lonza) and, after cisplatin removal, RPTECs were maintained for 24 h in the presence or absence of human UC-MSCs seeded 1:1 (30,000 cells/cm^2^) in the inserts of 24 mm transwell with 0.4 μm pore (Corning) in REBM basal medium (Lonza). In selected experiments, NHDF-Ad, used as control cells were seeded 1:1 (30,000 cells/cm^2^) in the inserts of 24 mm Transwell with 0.4 μm pore (Corning) in REBM basal medium (Lonza) at the same passages as UC-MSCs.

In additional experiments, RPTECs were treated with a non-toxic concentration^68^ of taxol 1 nM (Indena S.p.a, Milan, Italy). In our setting, the concentration of taxol used did not affect the number of living RPTECs exposed or not to cisplatin (Control: 54,412 ± 2582; Control + taxol: 55,931 ± 1180; Cisplatin: 38,235 ± 1274; Cisplatin + taxol: 37,255 ± 2137 cells/cm^2^; *n* = 3 independent experiments).

To prepare CM, UC-MSCs were incubated overnight in αMEM (Invitrogen) in serum free condition (15 ml per flask). The day after, medium was collected and centrifuged at 2000×*g* for 20 min at 4 °C to remove cellular debris. After centrifugation, supernatant was transferred into Amicon Ultra-15 centrifugal Filter Devices cut off 3 K (Merck Millipore) and centrifuged at 4000×*g* for 20 min to concentrate the volume of CM. Each aliquot of CM, obtained from 5 × 10^5^ UC-MSCs, was transferred into 1.5 ml tubes and frozen at −80 °C until use.

### Genome-wide differential gene expression analysis

Three biological replicates were performed for each group (Control, Cispl, and Cispl + UC-MSCs). Total RNA was extracted using the PureLink RNA Micro Scale Kit (Invitrogen), according to the protocol provided by the manufacturer. cDNA Libraries were prepared using the Ion AmpliSeq Transcriptome Human Gene Expression Kit (Thermo Fisher). Briefly, cDNA was first generated with SuperScript VILO cDNA Synthesis Kit from 10 ng of total RNA. Then, cDNA was amplified using Ion AmpliSeq technology to accurately maintain expression levels of all targeted genes. After target amplification, the resulting amplicons were ligated to barcode adapters. Libraries were then quantified by qPCR using the Ion Library Quantitation Kit (Thermo Fisher), and pooled in equimolar amounts. Template preparation (emulsion PCR) and sequencing on the Ion Proton Sequencer were performed at the CRIBI Biotechnology Center (Università degli Studi di Padova). Gene expression levels were provided as raw read counts. As the Ion AmpliSeq Transcriptome Human Gene Expression Kit covers each target gene with a single amplicon, normalization for transcript length bias is unnecessary. DEG analysis was performed on raw read counts using DESeq2 after discarding targets with <10 reads^69^. In addition, the RMRP gene target was discarded because it was highly expressed, accounting for ~3% of reads in all samples (Supplementary Fig. 3). DEGs, selected by their adjusted *P*-value and/or log2 fold change, were submitted to enrichment analysis using Enrichr^70^, focusing on gene ontology terms. Read counts were normalized by the regularized logarithm (rlog) method implemented in DESeq2 before PCA and to draw heat maps.

### Mitochondrial morphology and membrane potential detection

To evaluate mitochondrial morphology, living RPTECs were incubated with 250 nM fluorescent probe MitoTracker Red (#M7512; Thermo Fisher, Invitrogen) for 30 min, at 37 °C, 5% CO_2_. Nuclei were counterstained with NucBlue Live ReadyProbes (Hoechst; #R37605; Thermo Fisher, Invitrogen) according to the manufacturer’s protocol. At the end of probes incubations, living cells were examined by confocal inverted laser microscopy (LSM 510 Meta, Zeiss) and mitochondrial fragmentation was expressed as the % of Hoechst positive cells with fragmented mitochondria compared to elongate one identified by MitoTracker Red staining in 10 random fields per sample.

Mitochondria membrane potential was evaluated by exposing RPTECs to 5 μM JC-1 (#T3168; Thermo Fisher, Invitrogen) for 30 min, at 37 °C, 5% CO_2_. JC-1 exhibits potential-dependent accumulation in mitochondria indicated by a fluorescence shift from green (cytoplasm) to red (mitochondria). At the end of probe incubations, living cells were examined by confocal inverted laser microscopy (LSM 510 Meta, Zeiss) and the quantification of JC-1 red and green areas (Pixel^2^; Image J 1.40g software) was performed in 10 random fields per sample and mitochondria polarization was expressed as the ratio between red and green fluorescent area.

### MitoSOX assay in cultured cells

Mitochondrial ROS were measured using MitoSOX Red, a live-cell permeant mitochondrial superoxide (O2^•−^) indicator (#M36008; Thermo Fisher, Invitrogen). RPTECs were incubated with 5 μM MitoSOX for 30 min, at 37 °C, 5% CO_2_. Cells were collected by trypsinization, washed, and mitochondrial O2^•−^ was determined by FACS (FACS Canto, BD Biosciences, Milan, Italy). MitoSOX was excited by laser at 510 nm and data collected at FSC, SSC, 580 nm (FL2) channel. Data were expressed as MitoSOX mean fluorescence intensity (MFI) and % of MitoSOX fluorescent cells.

### Immunofluorescence analysis in cultured cells

At the end of incubations, RPTECs were fixed in 2% paraformaldehyde (PFA, Electron Microscopy Science, Hatfield, PA, USA) and 4% sucrose (Sigma-Aldrich) followed by permeabilization with 0.3% Triton X-100 (Sigma-Aldrich). Non-specific binding sites were blocked with 2% FCS, 2% BSA, and 0.2% bovine gelatine in PBS. Cells were incubated with a mouse anti-tubulin antibody (1:300; #T9026 clone DM1A; Sigma-Aldrich), a rabbit anti-SOD2 antibody (1:500; #AB10346; Merck Millipore), a rabbit anti-cleaved caspase-3 (Asp175) antibody (1:200; #9661; Cell Signaling, Danvers, MA, USA), a rabbit anti-phospho-histone H3 (Ser10) antibody (1:75; #9701; Cell Signaling), a rabbit anti-Kif5c antibody (1:50; #SAB1306219; Sigma-Aldrich), followed by incubation with a goat anti-rabbit Cy3-conjugated (1:80; Jackson ImmunoResearch Laboratories), or goat anti-mouse FITC-conjugated (1:80; Jackson ImmunoResearch Laboratories) secondary antibody, as appropriate. To study F-actin, fixed and permeabilized cells were incubated with rhodamine-phalloidin (20 U/ml; #R415; Thermo Fisher, Invitrogen). Nuclei were counterstained with DAPI (Sigma-Aldrich). Negative controls were obtained by omitting primary antibodies. After mounting with Dako (Agilent Technologies), cover slips were examined using a confocal inverted laser microscope (LSM 510 Meta, Zeiss). The quantification of the area corresponding to SOD2, cleaved caspase-3, and Kif5c staining was performed in 10 random fields per sample, expressed as MFI (Image J 1.40g software) and normalized for the number of DAPI positive cell nuclei (MFI/cell).

### Transfection experiments in cultured cells

To label mitochondria, RPTECs were transfected with a fusion construct of the leader sequence of E1 alpha pyruvate dehydrogenase fused with red (CellLight Mitochondria-RFP, BacMam 2.0; #C10601; Thermo Fisher, Invitrogen) or green fluorescent protein (CellLight Mitochondria-GFP, BacMam 2.0; #C10600; Thermo Fisher, Invitrogen) according to the manufacturer’s protocol. Briefly, RPTECs were seeded 20,000 cells/cm^2^ to reach a ~60% confluence and then two different populations of RPTECs were obtained by separately incubating overnight with 30 particles per cell, (PPC) of mitochondria-RFP or mitochondria-GFP. The day after transfection, RPTECs were washed twice, detached by trypsinization, and seeded 1:1 on cover slips in order to obtain an equal proportion of RPTECs with red- and green-tagged mitochondria. After 24 h, treatments were started and, at the end of incubations, cells were examined by confocal inverted laser microscopy (LSM 510 Meta, Zeiss). Nuclei were counterstained with NucBlue Live ReadyProbes (Hoechst; #R37605; Thermo Fisher, Invitrogen) according to the manufacturer’s protocol. Mitochondrial exchange among RPTECs was evaluated as the % of Hoechst positive cells with yellow mitochondria in 10 random fields per sample.

To silence SIRT3, RPTECs were transfected with 100 pmol Silencer Select predesigned siRNA human SIRT3 (siSIRT3; s23768; Thermo Fisher) or with control non-target siRNA (siNULL, Silencer Select Negative Control #2siRNA; Thermo Fisher) using lipofectamine 2000 reagent (Thermo Fisher, Invitrogen) according to the manufacturer’s protocol. After 24 h from transfection, cells were incubated with 5 μM cisplatin for 6 h, followed by 24 h incubation with control medium or human UC-MSCs in transwell, and then used for assessments of SIRT3 mRNA expression, analysis of mitochondrial fragmentation and polarization, mass, ATP production, and SOD2 expression. Co-staining of SOD and tubulin was also performed.

To overexpress SIRT3, RPTECs were seeded on 96 or 35 mm^2^ tissue culture plates at 70% confluence and were transfected with pCMV-hSIRT3-GFP (pSIRT3; Origene Technologies, Rockville, MD, USA) or pCMV6-AC-GFP (pNULL; Origene Technologies) using lipofectamine 2000 (Thermo Fisher, Life Technologies) as described in the manufacturer’s instructions. Briefly, cells were incubated with 5 µl of lipofectamine 2000 reagent and 2.5 μg of plasmid DNA mixed in 2 ml medium for 6 h followed by fresh medium for 24 h and then used for assessments of SIRT3 mRNA expression, analysis of mitochondrial mass, ATP production, and SOD2 expression. Co-staining of SOD and tubulin was also performed.

### ATP content assay

Whole renal tissues or human RPTECs were washed twice in normal 0.9% (w/v) sodium chloride solution and homogenized in a specific buffer for ATP assay (ATP Assay Kit, BioVision, Milpitas, CA, USA). The sample lysates were then centrifuged 12,000×*g* for 10 min at 4 °C, supernatants were harvested and total protein concentration was determined using DC assay (Bio-Rad Laboratories, Milan, Italy). Before ATP analysis, supernatants were quickly removed of proteins by deproteinizing Preparation Kit (BioVision) and then ATP content was analyzed according to the manufacturer’s protocol. The fluorescence intensities were determined by the multimode microplate reader TECAN Infinite M200 PRO (Tecan Group Ltd., Mannedorf, Schweiz) at an excitation wavelength of Ex 535 nm and emission wavelength of 587 nm. Results were normalized for total protein concentration of each sample.

### NAD^+^ content assay

Renal tissues or human RPTECs were washed twice in normal 0.9% (w/v) sodium chloride solution, homogenized in extraction buffer for NAD^+^ evaluation (NAD^+^/NADH Assay Kit, Abnova, Taipei City, Taiwan) and total protein concentration was determined using DC assay (Bio-Rad Laboratories). Samples were then processed for determination of NAD^+^ content by the multimode microplate reader TECAN Infinite M200 PRO (Tecan Group Ltd.) at 565 nm under controlled temperature for 15 min according to the manufacturer’s protocol. Results were normalized for total protein concentration of each sample.

### Mitochondria isolation and protein extraction

Mitochondria were isolated from renal tissue or human RPTECs using the Qproteome Mitochondria Isolation Kit (Qiagen, Hilden, Germany) according to the manufacturer’s protocol. For total protein extraction, excised renal tissues or human RPTECs were washed twice in normal 0.9% (w/v) sodium chloride solution and lysed by homogenization in CelLytic MT (Sigma-Aldrich) or sonication in mammalian cell lysis buffer (Sigma-Aldrich), respectively. Both lysis buffers were supplemented with protease inhibitor cocktail (Sigma-Aldrich) containing 104 mM AEBSF at 80 μM Aprotinin, 4 mM Bestatin, 1.4 mM E-64, 2 mM Leupeptin, and 1.5 mM Pepstatin A. After homogenization, the sample lysates were centrifuged 16,000×*g* for 10 min at 4 °C and protein concentration of both isolated mitochondria and total protein extracts was determined using DC assay (Bio-Rad Laboratories).

### Citrate synthase activity

Equal amounts of proteins (10 μg) from total extracts obtained by renal tissues or human RPTECs were analyzed with Citrate Synthase Activity Kit (Sigma-Aldrich) according to the manufacturer’s protocol. The citrate synthase activity was determined by the multimode microplate reader TECAN Infinite M200 PRO (Tecan Group Ltd.) at 412 nm under controlled temperature on a kinetic program for 1.5 min every 10 s.

### Western blot analysis

Equal amounts of 20 or 30 μg proteins from total extracts or isolated mitochondria obtained by renal tissues or human RPTECs were separated on 7.5–12% sodium dodecyl sulfate–polyacrylamide gel electrophoresis under reducing conditions and transferred to nitrocellulose membranes (Bio-Rad Laboratories). After blocking with 5% BSA (Sigma-Aldrich) in tris-buffered saline (TBS) supplemented with 0.1% Tween-20 (Sigma-Aldrich), membranes were incubated with the following antibodies: rabbit anti-SIRT3 (1:1000; #2627; Cell Signaling), rabbit anti-SIRT3 antibody (1:1000; #ab86671; Abcam) rabbit anti-Kif5c (1:500; #HPA035210; Sigma-Aldrich), and rabbit anti-acetyl lysine (1:1000; #9441; Cell Signaling). On the same membranes, mouse anti-GAPDH (1:1000; #TA802519 clone 2D9; Origene Technologies) and mouse anti-VDAC (1:1000; #ab61273; Abcam), were used as sample loading controls for evaluation of protein expression in total extract and isolated mitochondria, respectively. Acetylated tubulin expression was evaluated as the ratio between mouse anti-acetylated tubulin (1:1000; #T7451 clone 6-11B-1; Sigma-Aldrich) and total mouse anti-tubulin (1:1000; #T9026 clone DM1A; Sigma-Aldrich), after stripping of the same membrane. The signals were visualized on Odyssey FC Imaging System (LiCor, Lincoln, Nebraska, USA) by infrared (IR) fluorescence using a goat anti-rabbit IRDye 680LT (1:1000; LiCor) or a goat anti-mouse IRDye 800CW (1:1000; LiCor) secondary antibody, as appropriate. Bands were quantified by densitometry using the Image Studio Lite 5.0 (LiCor) software. All uncropped western blots can be found in Supplementary Fig. 9.

### General methods and statistical analysis

The sample size of the in vivo studies was estimated to be at least four mice per group to detect the expected difference with an 80% power (unpaired *t*-test, two-sided; *α* = 0.05), based on our previous published data^28^ in which mean ± SD of BUN levels were 122 ± 24 mg/dl in cisplatin mice given saline compared to 58 ± 24 mg/dl in cisplatin mice given MSCs. For in vitro experiments, the sample size was estimated to be at least three independent experiments to detect the expected difference with an 80% power (unpaired *t*-test, two-sided; *α* = 0.05), based on our previous published data^20^ in which mean ± SD of mitochondrial polarization ratio was 5.31 ± 0.25 in control RPTECs compared to 0.35 ± 0.25 in cisplatin-treated RPTECs. Samples/animals were randomly allocated to the experimental groups and no inclusion/exclusion criteria were used. In vivo studies in terms of renal function, histology, and SEM/TEM analysis were performed in a single-blind fashion.

Data are expressed as mean ± SEM of biological replicates. Data analysis was performed using the computer software Prism (GraphPad Software, Inc.). Comparisons were made using two-sided unpaired Student’s *t* test or two-sided ANOVA with the Bonferroni post hoc test as appropriate. Levene’s test was used to assess homogeneity of variance between the groups. Statistical significance was defined as *P* < 0.05.

All statistical analysis relative to DEGs were carried out in R (R Core Team (2016). R: A language and environment for statistical computing. R Foundation for Statistical Computing, Vienna, Austria. URL: https://www.R-project.org/.

### Study approval

The IRCCS—Istituto di Ricerche Farmacologiche Mario Negri adheres to the principles set out in the following laws, regulations, and policies governing the care and use of laboratory animals: Italian Governing Law (D.lgs 26/2014; Authorization n.19/2008-A issued March 6 2008 by Ministry of Health); Mario Negri Institutional Regulations and Policies providing internal authorization for persons conducting animal experiments (Quality Management System Certificate—UNI EN ISO 9001:2008—Reg. No. 6121); the NIH Guide for the Care and Use of Laboratory Animals (2011 edition) and EU directives and guidelines (EEC Council Directive 2010/63/UE). The Statement of Compliance (Assurance) with the Public Health Service (PHS) Policy on Human Care and Use of Laboratory Animals has been recently reviewed (9/9/2014) and will expire on September 30 2019 (Animal Welfare Assurance #A5023-01).

### Data availability

The authors declare that all data supporting the findings of this study are available within the article and its supplementary information files or from the corresponding author upon reasonable request. Genome-wide differential gene expression analyses have been deposited in the NCBI database under the accession code GSE98602.

## Electronic supplementary material

Supplementary Information

Supplementary Data 1

Supplementary Data 2

Supplementary Data 3
