# Supplementary material for: Human mesenchymal stromal cells transplanted into mice stimulate renal tubular cells and enhance mitochondrial function
Source: Nat Commun. 2017 Oct 17;8:983. doi: 10.1038/s41467-017-00937-2 (PMC5754365; doi:10.1038/s41467-017-00937-2)
Supplement: Supplementary file 1 — Supplementary Information [file 41467_2017_937_MOESM1_ESM.pdf]

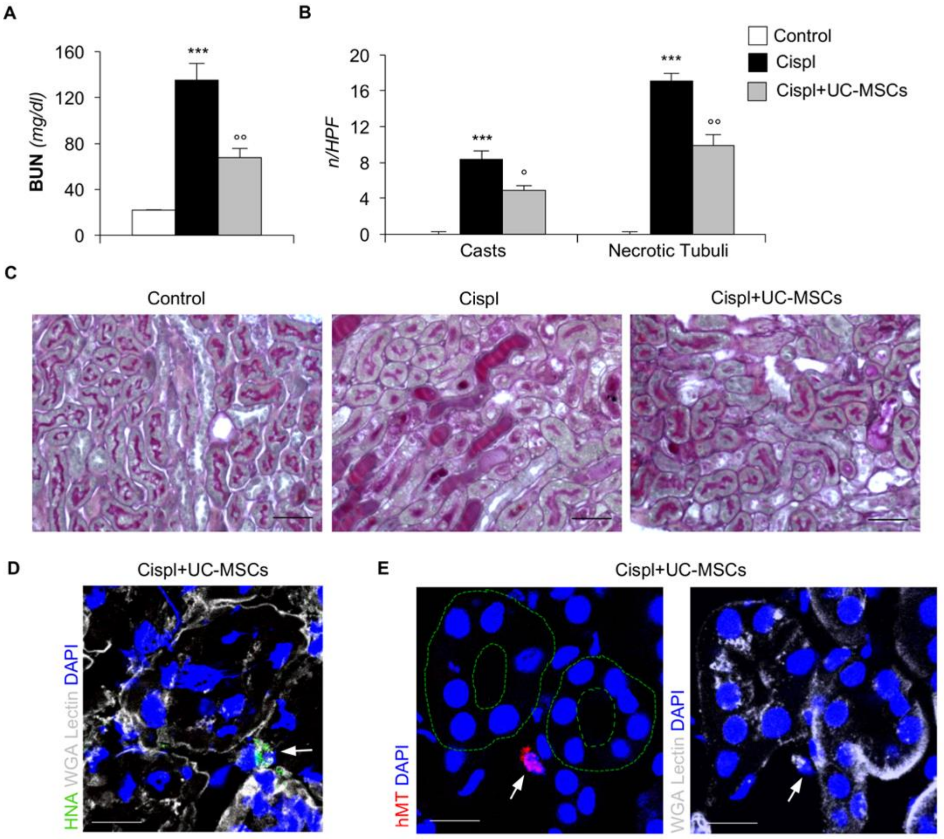

Supplementary Figure 1. UC-MSCs engraft the kidney and exert renoprotective effects in mice with AKI
(A) Renal function assessed as blood urea nitrogen (BUN) in control mice and cisplatin-treated mice receiving saline or human UC-MSCs at 4 days (n=6 mice *per* group). ***P<0.001 *vs* Control and °°P<0.01 *vs* Cispl using ANOVA corrected with Bonferroni coefficient. (B) Quantification of renal histological changes in control (n=6), and cisplatin-treated mice receiving saline (n=5) or human UC-MSCs (n=6) at 4 days evaluated as number (n) of casts and necrotic tubules per high power field (HPF). ***P<0.001 *vs* Control and °P<0.05, °°P<0.01 *vs* Cispl using ANOVA corrected with Bonferroni coefficient. (C) Representative images of histological changes in control, and cisplatin-treated mice receiving saline or human UC-MSCs at 4 days. Scale bar 50 μm. (D) Representative image of renal tissue of cisplatin-treated mouse at 4 days showing engrafted human UC-MSC stained for human nuclear antigen (HNA, green). WGA lectin labeled renal structures (white) and nuclei are counterstained with DAPI (blue). Scale bar 10 μm. (E) Representative images of the same renal section of cisplatin-treated mouse at 4 days showing UC-MSC labeled with human mitochondria antibody (hMT red; left panel. Dotted lines visualize tubular profiles) or WGA lectin (white, right panel). Nuclei are counterstained with DAPI (blue). Scale bar 10 μm. Data in panels A-B are expressed as mean ± SEM.

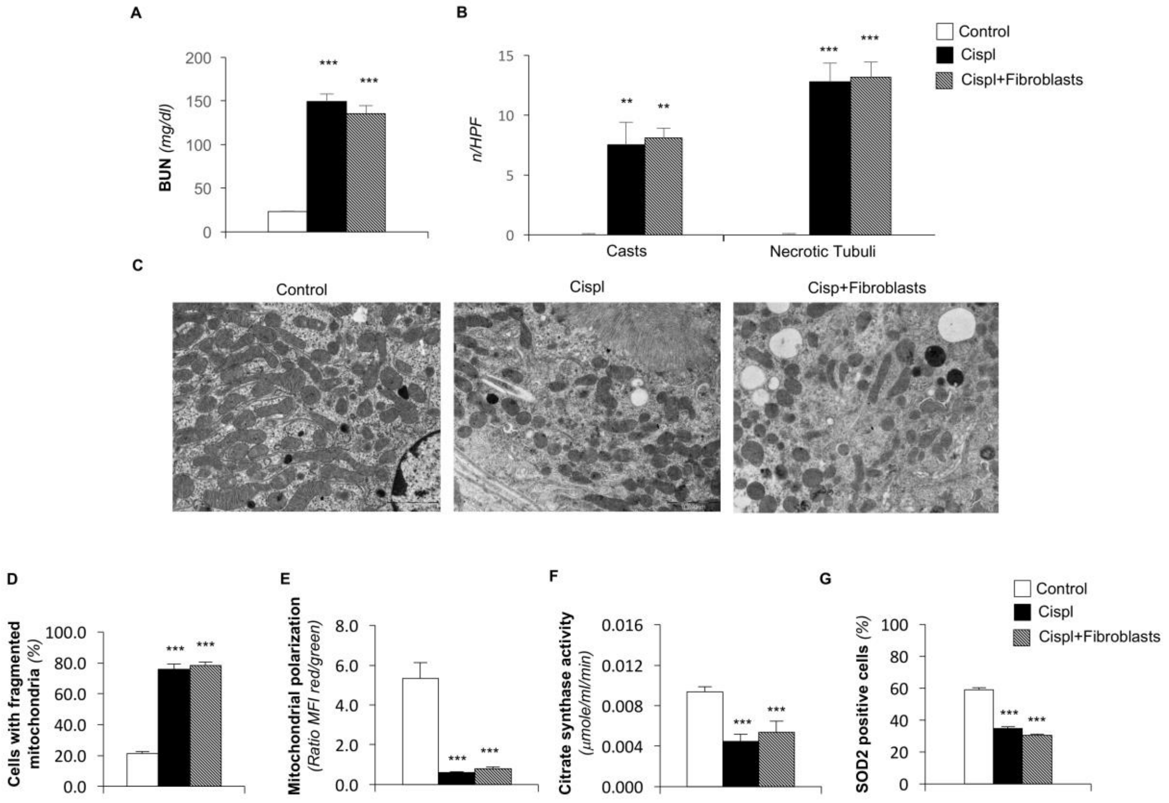

Supplementary Figure 2. Human fibroblasts fail to reduce renal tubular injury in mice with AKI
(A) Renal function assessed as blood urea nitrogen (BUN) in control mice and cisplatin-treated mice receiving saline or human dermal fibroblasts at 4 days (n=4 mice *per* group). ***P<0.001 *vs* Control, ANOVA corrected with Bonferroni coefficient. (B) Quantification of renal histological changes in control and cisplatin-treated mice receiving saline or human fibroblasts at 4 days evaluated as number (n) of casts and necrotic tubules per high power field (HPF) (n=4 mice *per* group). **P<0.01 and ***P<0.001 *vs* Control, ANOVA corrected with Bonferroni coefficient. (C) Representative transmission electron micrographs of the mitochondrial ultrastructure in proximal tubular cells of control and cisplatin mice given saline or human dermal fibroblast at 4 days (n=4 mice *per* groups). Scale bar 2000 nm. (D) *In vitro*, quantification of mitochondrial fragmentation assassed by MitoTracker staining in control RPTECs, cisplatin-treated RPTECs alone or co-cultured with human dermal fibroblasts (n=4 independent experiments). ***P<0.001 *vs* Control using ANOVA corrected with Bonferroni coefficient. (E) Quantification of mitochondrial membrane potential (red/green florescent area) visualized by JC-1, a dye sensitive to mitochondrial membrane potential ($\Delta\Psi m$) changes that shifts the emission spectrum from red (mitochondrial distribution) to green (cytoplasmic distribution) in control RPTECs, cisplatin-treated RPTECs alone or co-cultured with human dermal fibroblasts (n=4 independent experiments). ***P<0.001 vs Control using ANOVA corrected with Bonferroni coefficient. (F) Citrate synthase activity, reflecting mitochondrial mass, assessed in control RPTECs, cisplatin-treated RPTECs alone or co-cultured with human dermal fibroblasts (n=6 independent experiments). ***P<0.001 vs Control using ANOVA corrected with Bonferroni coefficient. (G) Quantification of SOD2 protein expression by immunofluorescence analysis in control RPTECs, cisplatin-treated RPTECs alone or co-cultured with human dermal fibroblasts (n=4 independent experiments). ***P<0.001 *vs* Control using ANOVA corrected with Bonferroni coefficient. Data in panels A-B and D-G are expressed as mean ± SEM.

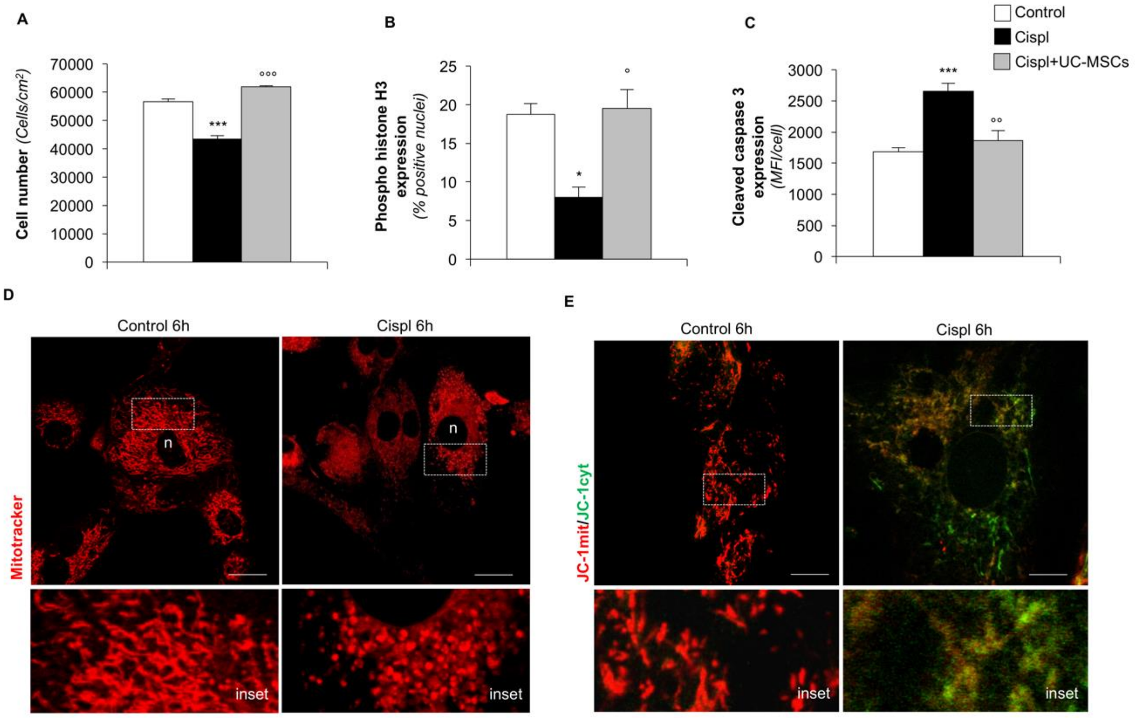

Supplementary Figure 3. UC-MSCs preserve proliferation and inhibit apoptosis in damaged tubular cells
(A) Quantification *in vitro* of the cell number in control RPTECs, cisplatin-treated RPTECs alone or co-cultured with human UC-MSCs (n=3 independent experiments). ***P<0.001 *vs* Control and °°°P<0.001 *vs* Cispl using ANOVA corrected with Bonferroni coefficient. (B) Quantification of proliferation expressed as the percentage of cells with nuclear staining of phospho histone H3 in control RPTECs, cisplatin-treated RPTECs alone or co-cultured with human UC-MSCs (n=3 independent experiments). *P<0.05 *vs* Control and °P<0.05 *vs* Cispl using ANOVA corrected with Bonferroni coefficient. (C) Quantification of apoptosis evaluated as cleaved caspase 3 staining and expressed as Mean Fluorescence Intensity (MFI) *per* cell in control RPTECs, cisplatin-treated RPTECs alone or co-cultured with human UC-MSCs (n=3 independent experiments). ***P<0.001 *vs* Control and °°P<0.01 *vs* Cispl using ANOVA corrected with Bonferroni coefficient. (D) Representative images showing mitochondrial morphology (visualized by staining with MitoTracker Deep Red) in control or cisplatin-treated RPTECs after 6h incubation (n=3 independent experiments). Enlarged details of mitochondrial fragmentation are shown in insets. n, nucleus. Scale bar 10 µm. (E) Mitochondrial membrane potential visualized by JC-1, a dye sensitive to mitochondrial ⊗m changes in control or cisplatin-treated RPTECs after 6h incubation (n=3 independent experiments). Depending on JC-1 distribution, the dye exhibit a shift from red (mitochondrial, JC-1mit) to green (cytoplasmic, JC-1cyt) in the emission spectrum. Enlarged details of mitochondrial depolarization are shown in insets. Scale bar 20 µm. Data in panels A-C are expressed as mean ± SEM.

A

B

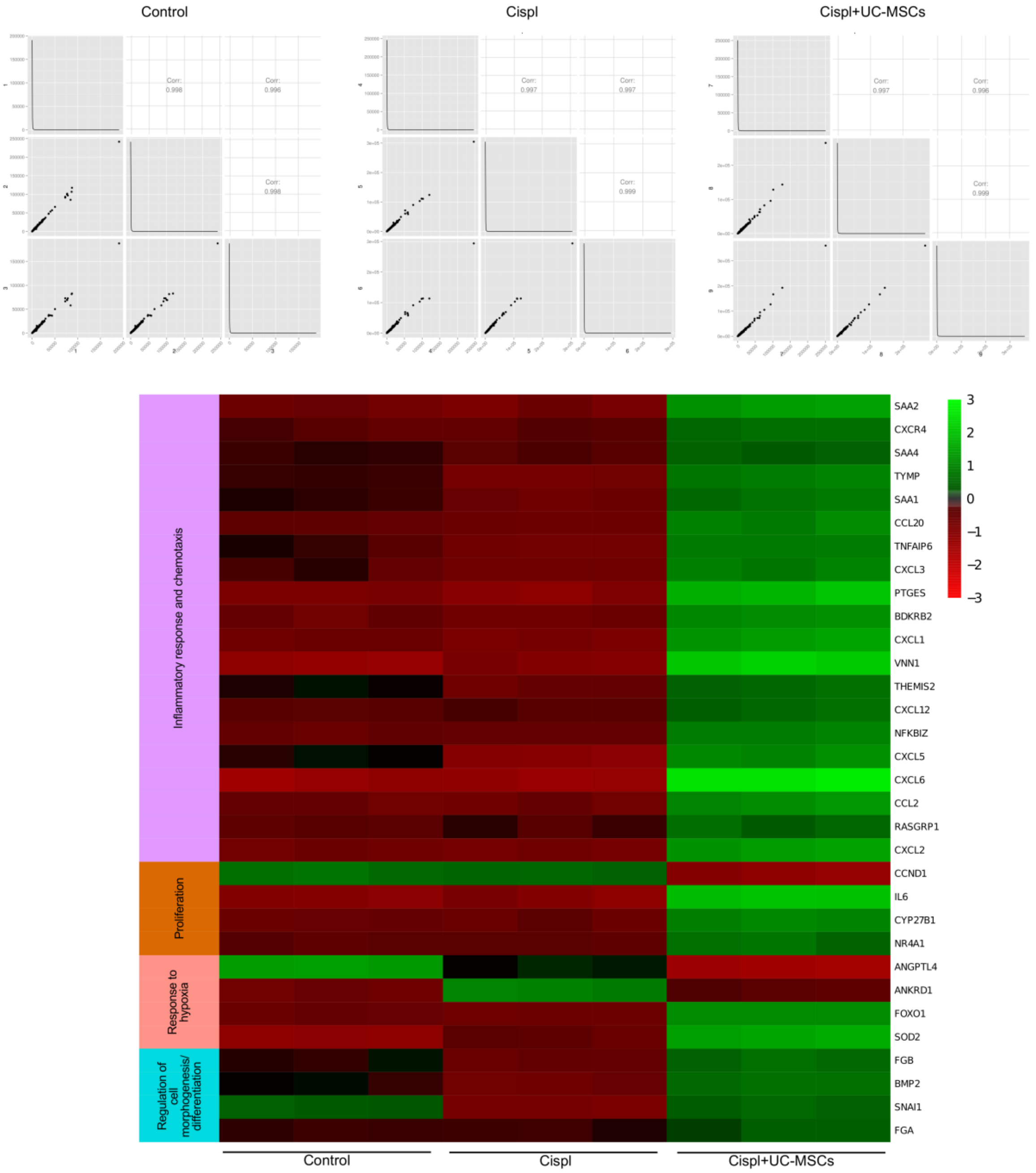

Supplementary Figure 4. Enrichment analysis of the most represented differentially expressed gene in tubular cells (A) Consistency of genome-wide differential gene expression analysis performed in three replicates of cultured RPTECs in resting conditions (Control; 1-3), exposed to cisplatin alone (Cispl; 4-6) or in co-culture with UC-MSCs (Cispl+UC-MSCs; 7-9). (B) Enrichment analysis of the selected top differentially expressed genes (DEGs) in RPTECs in resting conditions (Control), exposed to cisplatin alone (Cispl) or in co-culture with UC-MSCs (Cispl+UC-MSCs). Chemotaxis together with Proliferation, Regulation of cell morphogenesis/differentiation and Response to hypoxia, were among the four most strongly represented categories.

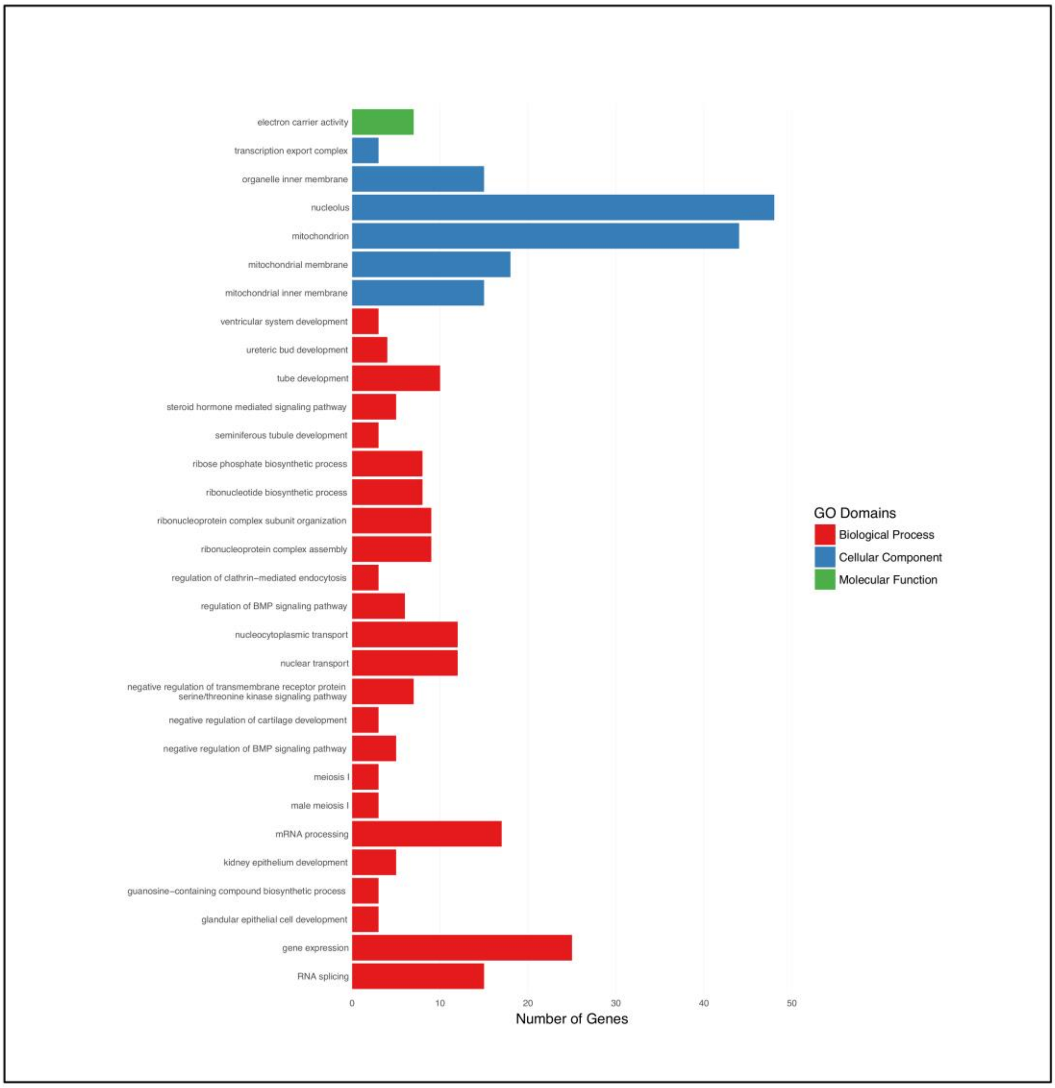

Supplementary Figure 5. Enrichment analysis of differentially expressed genes modulated by UC-MSCs in injured tubular cells
Enrichment analysis of the selected 409 differentially expressed genes (DEGs) exclusively modulated in cultured RPTECs by UC-MSCs. For each gene ontology (GO) domain, only GO terms that were significantly regulated (P<0.01) are depicted on the left. Bars represent the number of genes belonging to a given term that were differentially expressed.

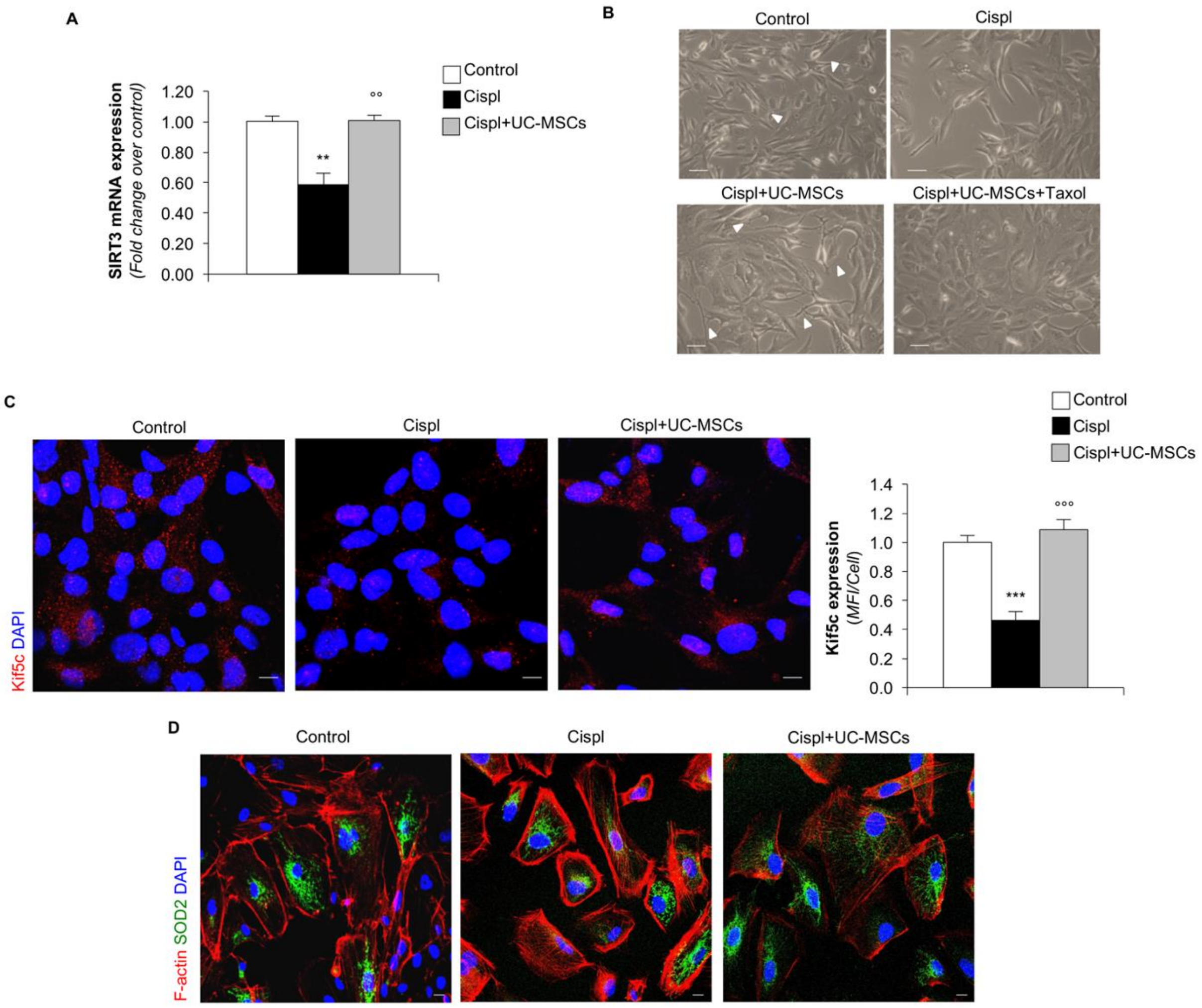

Supplementary Figure 6. UC-MSCs regulate SIRT3 and cytoplasmic protrusions among injured tubular cells
(A) Expression of SIRT3 mRNA by real-time PCR in control RPTECs, cisplatin-treated RPTECs alone or co-cultured with UC-MSCs (n=3 independent experiments). **P<0.01 *vs* Control and °°P<0.01 *vs* Cispl using ANOVA corrected with Bonferroni coefficient. (B) Representative phase contrast images showing the morphology of control RPTECs, cisplatin-treated RPTECs alone or co-cultured with UC-MSCs (n=4 independent experiments). In additional samples, cisplatin-treated RPTEC co-cultured with UC-MSCs were treated with taxol (n=4 independent experiments). Cytoplasmic protrusions connecting adjacent tubular cells are indicated by arrowheads. Scale bar 50 μm. (C) Representative images and quantification of Kif5c (red) protein expression by immunofluorescence analysis in control RPTECs, cisplatin-treated RPTECs alone or co-cultured with UC-MSCs (n=4 independent experiments). Nuclei are counterstained with DAPI (blue). Scale bar 10 μm. ***P<0.001 *vs* Control and °°°P<0.001 *vs* Cispl using ANOVA corrected with Bonferroni coefficient. (D) Representative images of immunofluorescence analysis of F-actin (red) and SOD2-labelled mitochondria (green) in control RPTECs, cisplatin-treated RPTECs alone or co-cultured with UC-MSCs (n=3 independent experiments). Nuclei are counterstained with DAPI (blue). Scale bar 10 μm. Data in panel A and C are expressed as mean ± SEM.

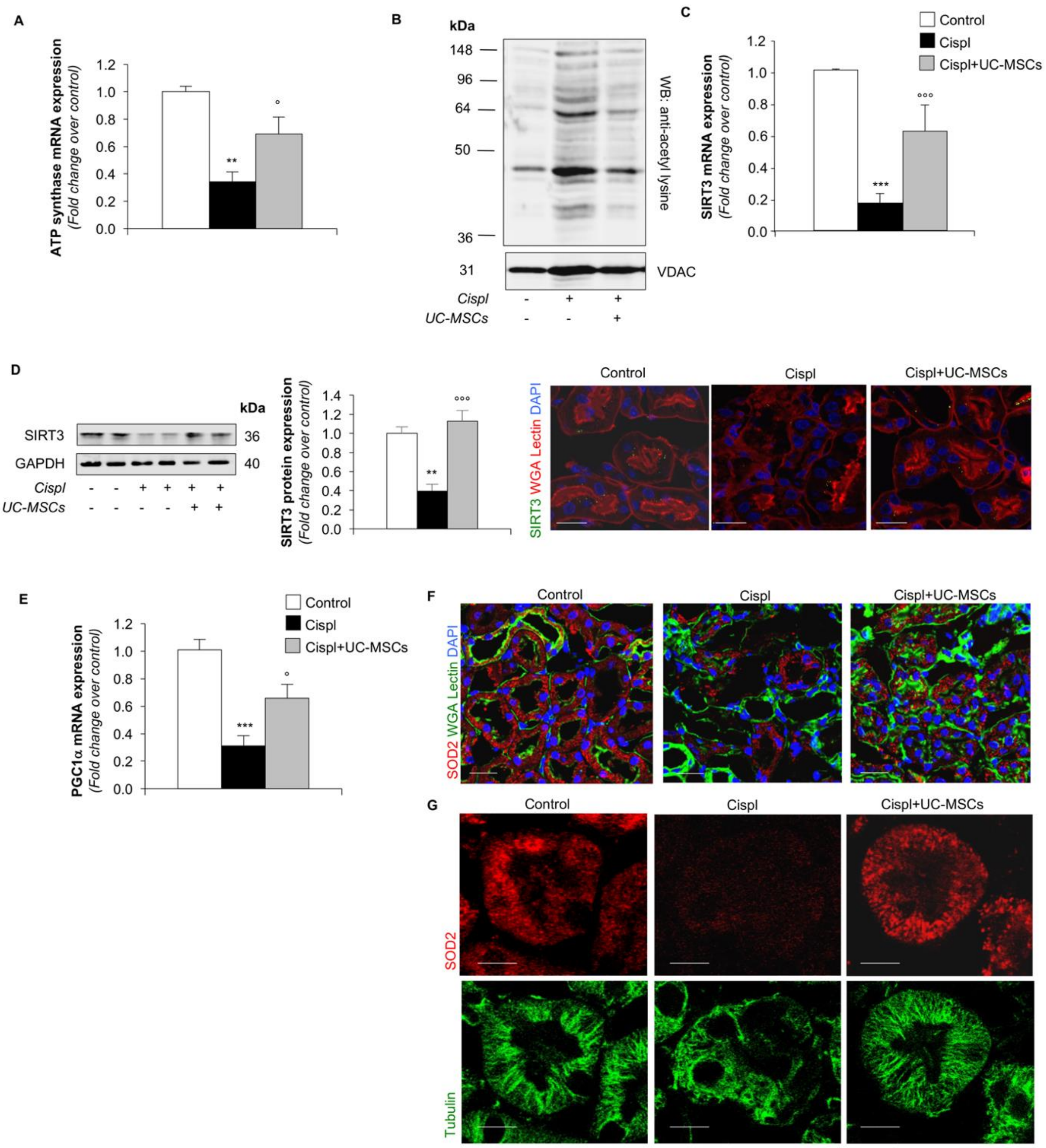

Supplementary Figure 7. UC-MSCs drive tubular recovery by regulating SIRT3 targets in mice with AKI
(A) Intrarenal expression of ATP synthase mRNA by real-time PCR in whole kidney of control (n=3 mice) and cisplatin mice given saline (n=4 mice) or UC-MSCs at 4 days (n=3 mice). **P<0.01 *vs* Control and °P<0.05 *vs* Cispl using ANOVA corrected with Bonferroni coefficient. (B) Representative Western blots of protein acetylation in mitochondria isolated from renal tissue of control and cisplatin-treated mice given saline or UC-MSCs at 4 days by using the anti-acetyl lysine antibody. VDAC protein expression was used as a sample loading control on the same membrane (n=3 mice *per* group). Molecular weights (kDa) are shown on the left. (C) Intrarenal expression of SIRT3 mRNA by real-time PCR in whole kidney of control (n=3 mice) and cisplatin mice given saline (n=5 mice) or UC-MSCs at 4 days (n=4 mice). ***P<0.001 *vs* Control and °°°P<0.001 *vs* Cispl using ANOVA corrected with Bonferroni coefficient. (D left panels) Western blot analysis and quantification of SIRT3 protein expression in whole kidney of control and cisplatin mice given saline or UC-MSCs at 4 days (n=4 mice *per* group). **P<0.01 *vs* Control and °°°P<0.001 *vs* Cispl using ANOVA corrected with Bonferroni coefficient. (D right panels) Representative images of SIRT3 (green) in control and cisplatin mice given saline or UC-MSCs at 4 days (n=3 mice *per* group). Nuclei are counterstained with DAPI (blue) and renal structures are stained with WGA lectin (red). Scale bar 10 μm. (E) Intrarenal expression of PGC1α mRNA by real-time PCR in whole kidney of control (n=3 mice) and cisplatin mice given saline (n=5 mice) or UC-MSCs at 4 days (n=4 mice). ***P<0.001 *vs* Control and °P<0.05 *vs* Cispl using ANOVA corrected with Bonferroni coefficient. (F) Representative images of immunofluorescence analysis of SOD2 (red) in control mice and cisplatin-treated mice receiving saline or UC-MSCs at 4 days (n=6 mice *per* group). Nuclei are counterstained with DAPI (blue) and renal structures are stained with WGA lectin (green). Scale bar 20 μm. (G) Representative single channel images of SOD2 expression (red) and tubulin distribution pattern (green) in control mice and cisplatin-treated mice receiving saline or UC-MSCs at 4 days referring to merge staining shown in Figure 7E. Scale bar 10 μm. Data in panels A and C-E are expressed as mean ± SEM.

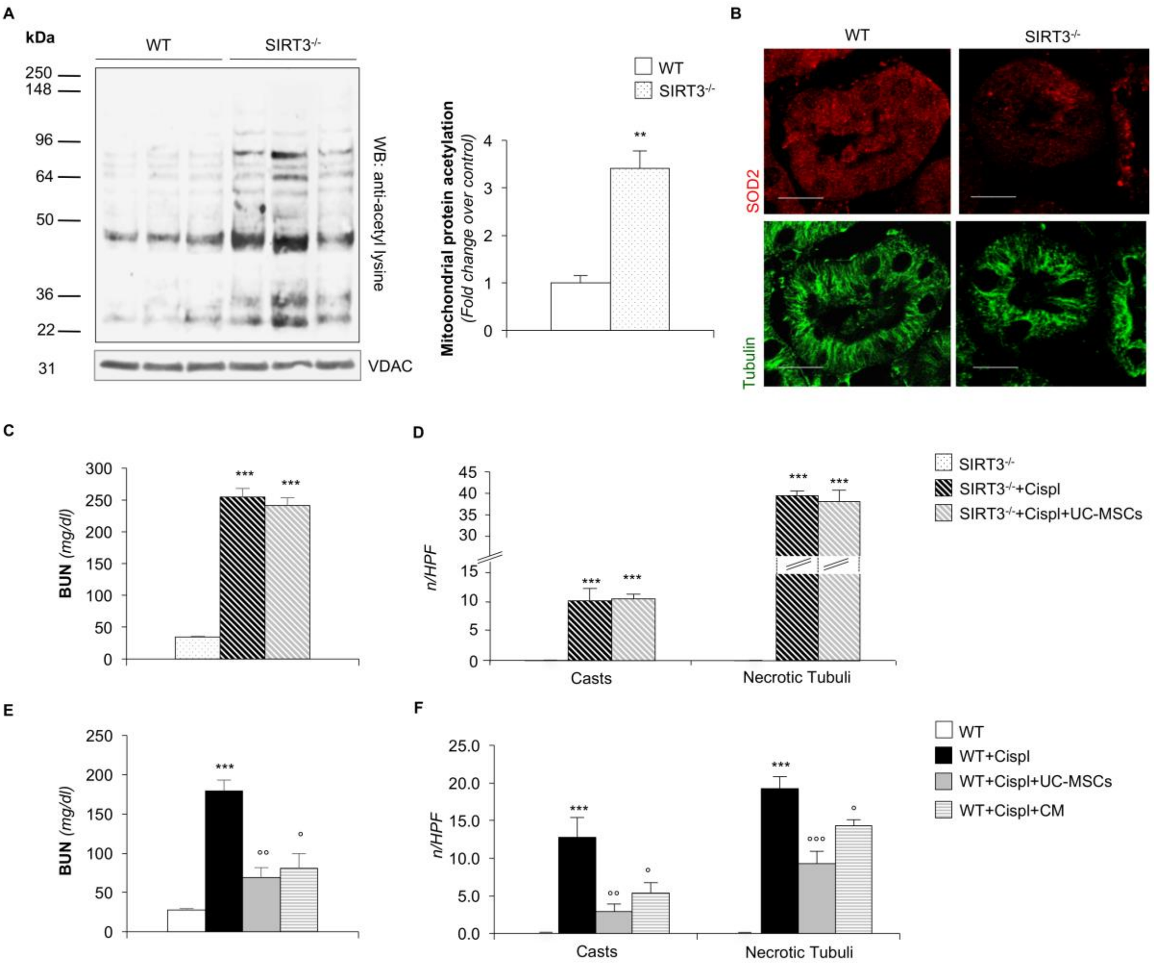

Supplementary Figure 8. UC-MSCs fail to induce renoprotection in SIRT3$^{-/-}$ mice with AKI

(A) Representative Western blots and densitometric analysis of protein acetylation in mitochondria isolated from renal tissue of untreated wild-type (WT) littermates and SIRT3 deficient (SIRT3$^{-/-}$) mice by using the anti-acetyl lysine antibody (n=3 mice *per* group). VDAC protein expression was used as a sample loading control on the same membrane. Molecular weights (kDa) are shown on the left. **P<0.01 *vs* WT using Student unpaired t-test. (B) Representative single channel images of SOD2 expression (red) and tubulin distribution pattern (green) in untreated WT mice and SIRT3$^{-/-}$ mice refferring to merge staining shown in Figure 7I. Scale bar 10 μm. (C) Renal function assessed as blood urea nitrogen (BUN) in untreated SIRT3$^{-/-}$ mice (n=4) and cisplatin-treated SIRT3$^{-/-}$ mice receiving saline (n=6) or human UC-MSCs (n=6) at 4 days. ***P<0.001 *vs* SIRT3$^{-/-}$ using ANOVA corrected with Bonferroni coefficient. (D) Quantification of renal histological changes in untreated SIRT3$^{-/-}$ mice (n=4), and cisplatin-treated SIRT3$^{-/-}$ mice receiving saline (n=4) or human UC-MSCs (n=6) at 4 days evaluated as number (n) of casts and necrotic tubuli per high power field (HPF). ***P<0.001 *vs* SIRT3$^{-/-}$ using ANOVA corrected with Bonferroni coefficient. (E) Renal function assessed as blood urea nitrogen (BUN) in untreated WT mice (n=5) and cisplatin-treated WT mice receiving saline (n=5), human UC-MSCs (n=6) or UC-MSCs-derived conditioned medium (CM, n=5) at 4 days. ***P<0.001 *vs* WT and °P<0.05 and °°P<0.01 *vs* WT+Cispl using ANOVA corrected with Bonferroni coefficient. (F) Quantification of renal histological changes in untreated WT mice (n=5) and cisplatin-treated WT mice receiving saline (n=5), human UC-MSCs (n=5) or UC-MSCs-derived conditioned medium (CM, n=5) at 4 days evaluated as number (n) of casts and necrotic tubules per high power field (HPF). ***P<0.001 *vs* WT and °P<0.05 and °°°P<0.001 *vs* WT+Cispl using ANOVA corrected with Bonferroni coefficient. Data in panels A and C-F are expressed as mean ± SEM.

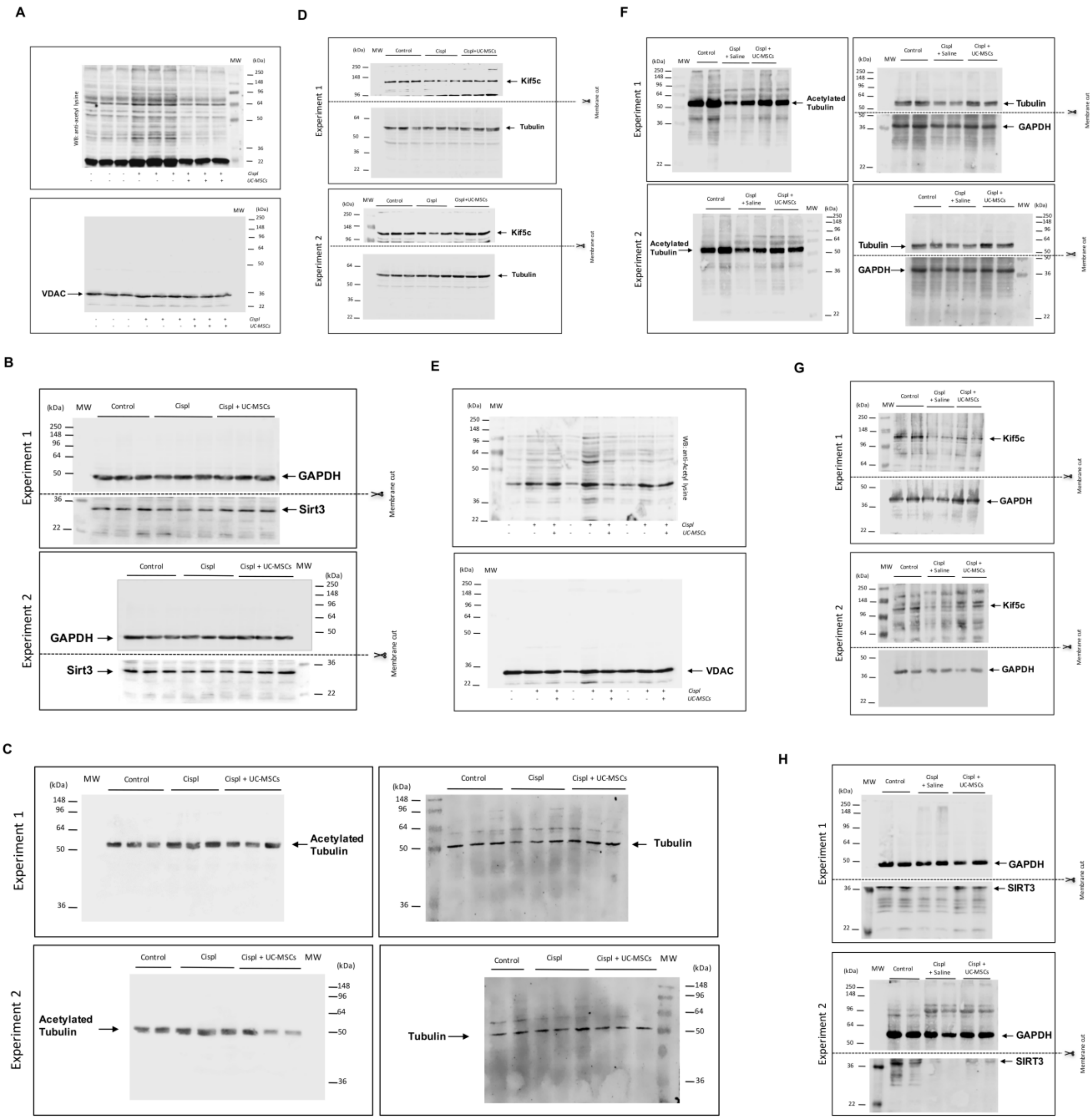

Supplementary Figure 9. Uncropped gels of all proteins analyzed in Western blot experiments

(A) Uncropped gels of protein acetylation and VDAC expression in isolated mitochondria from RPTECs shown in Figure 3C. (B) Uncropped gels of SIRT3 and GAPDH protein expression in RPTECs shown in Figure 3D. (C) Uncropped gels of tubulin expression, tubulin acetylation, and GAPDH in RPTECs shown in Figure 5A. (D) Uncropped gels of Kif5c and tubulin protein expression in RPTECs shown in Figure 5B. (E) Uncropped gels of protein acetylation and VDAC expression in isolated mitochondria from kidneys of NOD/SCID mice shown in Figure 7C and Supplementary Figure 7B. (F) Uncropped gels of tubulin expression, tubulin acetylation, and GAPDH in NOD/SCID renal extracts shown in Figure 7F and G. (G) Uncropped gels of Kif5c and GAPDH protein expression in NOD/SCID renal extracts shown in Figure 7H. (H) Uncropped gels of SIRT3 and GAPDH protein expression in NOD SCID renal extracts shown in Supplementary Figure 7D.
